# An observability and detectability analysis for non-linear uncertain CSTR model of biochemical processes

**DOI:** 10.1038/s41598-022-26656-3

**Published:** 2022-12-25

**Authors:** Mateusz Czyżniewski, Rafał Łangowski

**Affiliations:** 1 Department of Intelligent Control and Decision Support Systems, Gdańsk University of Technology, G. Narutowicza 11/12, 80-233 Gdańsk, Poland; 2 Digital Technologies Center, Gdańsk University of Technology, G. Narutowicza 11/12, 80-233 Gdańsk, Poland

**Keywords:** Engineering, Mathematics and computing

## Abstract

The problem of proving observability/detectability properties for selected non-linear uncertain model of biochemical processes has been addressed in this paper. In particular, the analysis of observability/detectability in the face of parametric and unstructured uncertainty in system dynamics transformed into unknown inputs, and unknown initial conditions has been performed. Various sets of system measured outputs were taken into account during the research. The considered biochemical processes were modelled as a continuous stirred tank reactor with the microbial growth reaction and microbial mortality with the aggregated substrate and biomass concentrations in aerobic phase. Classical tools based on differential geometry and the method of indistinguishable state trajectories (indistinguishable dynamics) were used to verify the properties of the system. The observability/detectability analysis was performed for nine cases covering a wide range of possible combinations of system measured outputs and unknown inputs. The obtained results of are crucial meaning for system state reconstruction (estimation), which involves the synthesis of state observers.

## Introduction

Nowadays, effective handling of a majority of industrial processes requires advanced control and monitoring algorithms. This requirement can be understood in many ways, such as meeting assumed control objectives, ensuring process safety, or the cost efficiency of the performed process. The algorithms used must enable this operation by covering fields such as control, monitoring, estimation, diagnostics, or optimisation. It undoubtedly involves the issue of access to the information about process variables, e.g., state variables, controlled output variables (signals), etc., of the system (plant) in which the process occurs. It is known, e.g., from operational practice that the access to these variables is limited. There are various reasons for this, and the most typical ones include the impossibility of measuring a given variable due to a lack of measuring devices (sensors), physical impossibility of installing a sufficient number of measuring devices in a given physical system, high cost of purchasing and exploiting measuring devices and low quality of the measurements provided due to measurement errors or measurement noise. Therefore, the missing information on, e.g., state variables may be completed by their estimates. Typically, the reconstruction (estimation) of the system state is based on the mathematical model of a given process and measurements of other available variables (system measured outputs). The typical tool used for system state estimation is a state observer.

A significant number of various state observer structures can be found in the literature. The choice of the type of state observer is mainly driven by the features of the system whose state is to be estimated. In this paper, a bioreactor in which biochemical processes take place, that is part of a wastewater treatment plant is considered^1^. From the point of view of mathematical modelling, which is an essential stage of system state reconstruction, two main groups of non-linear models of biochemical processes can be indicated. These include activated sludge models (ASMs) and balance models^2–7^. The ASM model family is seen as the most faithfully reflecting the behaviour of natural processes in a bioreactor^2,4,6^. Thus, they are primarily used for simulation purposes, e.g., to predict the run of a given process under certain operating conditions. On the other hand, these models are burdened with a significant degree of complexity and, therefore, it is not always possible to use them to synthesise a state observer or a control system. An alternative is to use balance models based on physical balance laws and aggregating certain individual fractions occurring in ASM models^2,4^. Unfortunately, the simpler structure of the balance models often entails the appearance of both parametric (structured) and unstructured uncertainty in them. The parametric uncertainty stems from inaccurate knowledge of the values of system parameters, while the unstructured uncertainty, in turn, usually holds unmodelled system dynamics (includes intended model simplifications). In this paper, a non-linear uncertain balance model of a biochemical processes is considered. It is worth adding that this kind of model belongs to a certain sub-class of the general class of affine non-linear dynamic systems^8,9^. In turn, typically taking uncertainty into account can be done through stochastic or deterministic approaches. The first approach requires reliable probabilistic models. To obtain such models, it is necessary to have sufficient data to guarantee the reliability of the assumed probability density functions. One of the most popular estimation methods using probabilistic uncertainty models also for measurement noise is estimation using the Kalman filter^10,11^. Observers based on Kalman filter or, especially, an extended version of it, i.e., extended Kalman filter are widely used in the state estimation of biochemical processes, also in a situation of not exactly known inputs^12–20^ . In contrast, the use of a deterministic approach generally leads to the synthesis of Luenberger-like observers^2,21–24^. For example, a set-membership approach to uncertainty modelling is used in the design of an interval observer^25^. It involves that an interval observer produces upper and lower envelopes bounding the reconstructed state variable^26–29^. Another way is to eliminate the uncertainty from the system dynamics by transferring it to an additional model component. From the point of view of the further state observer synthesis, this component is treated as some additional unknown input (UI). This methodology gives very good results and its application leads to so-called unknown input observers (UIOs)^30–38^. The UIOs include sliding mode observers (SMOs), which enable the generation of a point estimate of the reconstructed state variable under uncertainty impact. Due to their properties, e.g., the possibility of ’estimating’ the uncertainty, sliding mode observers have been widely used in biochemical systems, e.g.,^30,33,34,39–48^. However, it is important to be aware that SMOs are very sensitive to measurement noise, hence their implementation requires the use of appropriate filters. In the following sections of the paper some of the considerations take into account the possibility of further synthesis of the sliding mode observer. It is also worth adding that using of UIOs for estimation purposes in other applications than biochemical processes is widely addressed in the literature. For example, they are used in mechanical, electrical or water systems^49–55^.

It should be emphasised that the reconstruction of the system state associated with the synthesis of a given state observer is strictly related to the observability/detectability (asymptotic observability) properties of the system model. In general, when the particular input-output behaviour of the system is known, observability addresses the ability to exactly reconstruct the system state in a finite time-horizon, whereas detectability only enables estimating the system state in an asymptotic way^23,26,56–58^. It is also worth highlighting that there are many different notions of observability/detectability for non-linear systems and they are more difficult to prove in such systems than in linear systems^8,12,56,59–61^. Moreover, the mathematical model of a given system is only a certain, idealised representation of reality which primarily addresses the most essential features of the considered natural phenomena. Consequently, there is parametric and unstructured uncertainty. Furthermore, it is typical in the system that the initial conditions are not exactly known. Uncertainty can also burden input signals to the system, which is manifested by, e.g., the occurrence of mentioned unknown inputs. In this situation, the observability/detectability concepts become more complicated and extended. However, both of these two properties should be maintained despite the uncertainties involved.

Hence, the main aim of this work is to investigate the observability/detectability for selected non-linear model of biochemical processes in the presence of unknown inputs. A continuous stirred tank reactor (CSTR) with the microbial growth reaction and microbial mortality with the aggregated substrate and biomass (reactants) concentrations in aerobic phase is used as a cognitive bioreactor model^2,4^. The analysis presented takes into account various configurations of system measured outputs. A method of indistinguishable dynamics (indistinguishable system state trajectories) is used to prove these properties^56,59,62–64^. Moreover, the classical approach based on differential geometry tools is used to provide sufficient conditions for observability^8,56,59,63,65–68^. Several methodological aspects of the analysis carried out are indicated. To summarise, the main contributions of this paper are as follows:

- an observability/detectability analysis of the non-linear CSTR model of biochemical processes in the face of parametric and unstructured uncertainty and unknown initial conditions, and taking into account various sets of system measured outputs has been devised,
- a comprehensive discussion on performing simplification of the cognitive CSTR model for the observability/detectability analysis has been given,
- a method of indistinguishable dynamics and an approach based on differential geometry tools has been used during research, highlighting their essential features and aspects,
- the analysis has been presented for nine cases covering a wide range of choices of system measured outputs, along with the way how to eliminate uncertainty from system dynamics.

The paper is organised as follows. The background and problem statement are presented in Sections "Background" and "Problem statement", respectively. In Section "Cognitive CSTR model" the cognitive model of considered biochemical processes is given. Section "CSTR model for observability/detectability analysis purposes" includes derivation of the CSTR model for observability/detectability analysis purposes. A detailed observability/detectability analysis of the non-linear uncertain CSTR model is presented in Section The observability/detectability analysis of the CSTR model. The paper is concluded in Section Conclusions and completed with three Appendices.

## Background

In this Section, the background of the most important aspects of the paper is presented. It includes defining the considered affine uncertain non-linear dynamic system, describing possible ways of analysing observability/detectability and investigating the problem of so-called ’bad inputs’.

### Description of affine non-linear system

Considering ${\mathbb {R}}^\mathrm{n}$ as the *n*–dimensional vector space over a real number field ${\mathbb {R}}$, a multiple-input single-output (MISO) affine non-linear system $\Sigma _\mathrm {\nu }$ can be defined as follows^8,9^:

1 $$\begin{aligned} \Sigma _\mathrm {\nu }: {\left\{ \begin{array}{ll} \dot{\varvec{\nu }}(t) &{}= \varvec{\varphi }(\varvec{\nu }(t),t) + \sum \limits _{i_\mathrm{p}=1}^{p} \varvec{\vartheta }_{i_\mathrm{p}}(\varvec{\nu }(t))u_{i_\mathrm{p}}(t)\\ \varvec{\nu }\left( t_0 \right) &{}= \varvec{\nu }_0\\ y(t) &{}= h(\varvec{\nu }(t)) \end{array}\right. }, \end{aligned}$$

where: $\dot{(\cdot )}$ stands for the derivative with respect to *t*; $t \in {\mathbb {T}} = {\mathbb {R}}_{+} \cup \{0\}$ is the time instant, ${\mathbb {R}}_{+}$ denotes a positive part of ${\mathbb {R}}$; $\forall t \in {\mathbb {T}} :~ \varvec{\nu }(t) \in {\varvec{\mathcal {M}}} \subset {\mathbb {R}}^{\mathrm{n}}$ is the vector of state variables, which coincide with globally defined cubic coordinates, $\varvec{{\mathcal {M}}}$ is a (connected) differentiable manifold with $\varvec{\mathcal {C}}^{\infty }(\cdot )$ structure of dimension *n*; $\forall t \in {\mathbb {T}} :~ \Vert u_{i_{\mathrm{p}}}(t) \Vert _{\infty }=\sup \left\{ \mid u_{i_{\mathrm{p}}}\mid :~ t \in {\mathbb {T}} \right\} \le u^\mathrm{bound}_{i_{\mathrm{p}}} <\infty , i_{\mathrm{p}}= \overline{1,p}$ denotes the $i_{\mathrm{p}}$th exogenous input belonging to $\forall t \in {\mathbb {T}} :~ \varvec{u}(t) \in {\mathbb {U}}_\mathrm{p} \subset {\mathbb {R}}^{p}$; $\varvec{\nu }_0$ signifies the vector of initial conditions; $t_0$ denotes the initial time equal to zero; $\forall t \in {\mathbb {T}} :~ y(t) \in {\mathbb {Y}}_\mathrm{q} \subset {\mathbb {R}}^{q}$ is the vector of system measured outputs; $\forall t \in {\mathbb {T}} :~ \varvec{\varphi } \in \varvec{\mathcal {C}}^{\infty }(\varvec{\mathcal {M}}) :~ \varvec{\mathcal {M}} \times {\mathbb {T}} \rightarrow \mathrm{T}\varvec{\mathcal {M}}$,   $\forall t \in {\mathbb {T}} :~ \varvec{\vartheta }_{i_\mathrm{p}} \in \varvec{\mathcal {C}}^{\infty }(\varvec{\mathcal {M}}) :~ \varvec{\mathcal {M}} \rightarrow \mathrm{T}\varvec{\mathcal {M}}$,   $\forall t \in {\mathbb {T}} :~ h :~ \varvec{\mathcal {M}} \rightarrow {\mathbb {Y}}_\mathrm{q}$ are the smooth maps; $\mathrm{T}(\cdot )$ stands for the tangent bundle of vector field; $\times$ denotes a Cartesian product.

It should be added that the system $\Sigma _\mathrm {\nu }$ is claimed as complete, i.e., the system state trajectories $\varvec{\nu }(t)$ are defined for every $t \in {\mathbb {T}}$ and every initial condition $\varvec{\nu }_{\mathrm{0}}$ and for all exogenous inputs which belong to their particular sets.

In turn, it is assumed that the internal dynamics of $\Sigma _\mathrm {\nu }$ represented by $\varvec{\varphi }(\varvec{\nu }(t),t)$ is not exactly known due to parametric and unstructured uncertainty. Due to uncertain nature of $\varvec{\varphi }(\varvec{\nu }(t),t)$, the new $\hat{\varvec{\varphi }}(\varvec{\nu }(t)) \in \varvec{\mathcal {C}}^{\infty }(\cdot ) :~ \varvec{\mathcal {M}} \rightarrow \mathrm{T}\varvec{\mathcal {M}}$, which is the exactly known (independent of parametric and unstructured uncertainty) assessment of $\varvec{\varphi }(\varvec{\nu }(t),t)$ for some sub-class of affine non-linear systems can be introduced as:

2 $$\begin{aligned} \varvec{\rho }(\varvec{\nu }(t))\Delta (\varvec{\nu }(t),t) \triangleq \varvec{\varphi }(\varvec{\nu }(t),t) - \hat{\varvec{\varphi }}(\varvec{\nu }(t)), \end{aligned}$$

where $\forall t \in {\mathbb {T}} :~ \Delta (\varvec{\nu }(t),t) :~ \varvec{\mathcal {M}} \times {\mathbb {T}} \rightarrow {\mathbb {W}} \subset {\mathbb {R}}$ with $\Vert \Delta (\varvec{\nu }(t),t) \Vert = \text {max} \left\{ \mid \Delta (\varvec{\nu }(t),t) \mid :t \in {\mathbb {T}} \right\} \le {\overline{\Delta }} <\infty$; $\forall t \in {\mathbb {T}} :~ \varvec{\rho } \in \varvec{\mathcal {C}}^{\infty }(\cdot ) :~ \varvec{\mathcal {M}} \rightarrow \mathrm{T}\varvec{\mathcal {M}}$ is the smooth map.

Given the assumption expressed by (2), the following form of $\Sigma _\mathrm {\nu }$, which is a model of some sub-class of affine non-linear systems, can be written as:

3 $$\begin{aligned} \Sigma _\mathrm{d}: {\left\{ \begin{array}{ll} \dot{\varvec{\nu }}(t) &{}= \hat{\varvec{\varphi }}(\varvec{\nu }(t)) + \sum \limits _{i_\mathrm{p}=1}^{p} \varvec{\vartheta }_{i_\mathrm{p}}(\varvec{\nu }(t))u_{i_\mathrm{p}}(t) + \varvec{\rho }(\varvec{\nu }(t))\Delta (\varvec{\nu }(t),t)\\ \varvec{\nu }\left( t_0 \right) &{}= \varvec{\nu }_0\\ y(t) &{}= h(\varvec{\nu }(t)) \end{array}\right. }. \end{aligned}$$

The component $\Delta (\varvec{\nu }(t),t)$ may be considered as an additional input to system $\Sigma _\mathrm{d}$ bounded by ${\overline{\Delta }}$ . Moreover, taking into account (2) and interpreting $\varvec{\rho }$ as a vector parameterising the uncertainty of the dynamics of the original system (1), the component $\varvec{\rho }(\varvec{\nu }(t))\Delta (\varvec{\nu }(t),t)$ models the parametric and unstructured uncertainty and thus represents the (parameterised) unknown inputs of the system (3). This makes the vector of state variables $\varvec{\nu }(t)$ independent of the impact of uncertainty. This transformation provides very interesting properties since even if the uncertainty is not the physically occurring unmeasured signal, introducing the new variable as the difference between real internal dynamics burdened by some uncertainties and known estimation gives the chance of its compensation by method addressed to UI-related systems. The unknown inputs expressed by $\Delta (\varvec{\nu }(t),t)$ are depended on $\varvec{\nu }(t)$; however, they are not considered as tangent mapping but as certain distinct signals which coincide with the field $\varvec{\rho }(\varvec{\nu }(t))$. Imposing this particular consideration of uncertainty has two strong advantages. The first advantage is associated with the possibility of performing the observability/detectability analysis on the perfect model of the system belonging to this sub-class of non-linear affine systems based on differential geometry tools. The second one makes a way of estimation of uncertain part of the system dynamics by a certain additional component in the structure of the state observer, e.g.,^30,41,46,69–74^.

It is worth adding that there may be situations in which, due to the complex structure of a given model of the system under consideration, it is preferable to decompose the system dynamics before performing transformations eliminating the uncertainty. The idea of such a decomposition is included in Appendix A.

### Observability/detectability analysis for non-linear systems

As it has been mentioned, there are many different notions of observability/detectability for non-linear systems. The classical approach to proving the observability of a non-linear system is based on differential geometry tools^8,56,59,63,65–68^. It is associated with using ’observability maps’ expressed as the set of subsequent Lie derivatives of the system measured outputs along the drift vector field. By checking the injectivity of the Lie derivatives , e.g., by applying the inverse function theorem to the map’s Jacobi matrix (checking its invertibility ), or investigating the positiveness of principal minors of the Jacobi matrix, it is possible to prove whether the system is observable or not^8,32,56,59,73,75^. This approach is also called ’observability rank condition’ and in detail is associated with local properties of the manifold $\varvec{\mathcal {M}}$. Due to the fact that this particular methodology is essentially based on the theory of differential geometry and the theory of differential equations, the observability notion is extended and narrowed to local, weak and local weak observability^8,32,56^. Therefore, this approach generally provides only sufficient conditions for observability. This is due to that the observability can be given only for some time interval, which is dependent on factors associated with the properties of system dynamics^8,56,65,68,76^. Hence, for the considered system (3) the observability map (’observation space’) $\mathcal {O} \in \mathcal {C}^{\infty }(\varvec{\mathcal {M}})$ yields^8,56,59,65^:

4 $$\begin{aligned} \mathcal {O} = \begin{bmatrix} h \\ L_{\hat{\varphi }}h \\ \vdots \\ L_{\hat{\varphi }}^\mathrm {n-1} h \end{bmatrix}, \end{aligned}$$

where $L_{(\cdot )}^{(\cdot )}(\cdot )$ denotes the Lie derivative^8,77^.

The observability matrix is derived by exterior differentiation of $\mathcal {O}$ concerning the state vector $\varvec{\nu }(t)$. In this single measured output system, it is equal to the following observability co-distribution after imposing particular coordinates of $\varvec{\nu }(t)$:

5 $$\begin{aligned} d\mathcal {O} = \text {span} \left\{ dh, ~ dL_{\hat{\varphi }}h, ~, \cdots , ~ d L_{\hat{\varphi }}^\mathrm {n-1} h :~ h, ~ L_{\hat{\varphi }} h, ~, \cdots , ~ L_{\hat{\varphi }}^\mathrm {n-1} h \in \mathcal {O} \right\} , \end{aligned}$$

where $d(\cdot )$ stands for exterior differentiation, which is invariant with respect to the fields $\hat{\varvec{\varphi }}(\varvec{\nu }(t))$, $\varvec{\vartheta }_{i_\mathrm{p}}(\varvec{\nu }(t))$ and $\varvec{\rho }(\varvec{\nu }(t))$^8,77^.

Thus, the observability matrix which is equivalent to the Jacobi matrix is as follows:

6 $$\begin{aligned} \partial _{\mathrm {\varvec{\nu }}}\varvec{\Phi }(\varvec{\nu }(t)) = \begin{bmatrix} dh \\ dL_{\hat{\varphi }}h \\ \vdots \\ d L_{\hat{\varphi }}^\mathrm {n-1} h \end{bmatrix} = \begin{bmatrix} \nabla h \\ \nabla L_{\hat{\varphi }}h \\ \vdots \\ \nabla L_{\hat{\varphi }}^\mathrm {n-1} h \end{bmatrix}, \end{aligned}$$

where $\nabla (\cdot )$ denotes the gradient operator.

If $d\mathcal {O}$ spans the whole manifold $\varvec{\mathcal {M}}$ (excluding singular points), which means that the rank of the observability matrix $\partial _{\mathrm {\varvec{\nu }}} \varvec{\Phi }(\varvec{\nu }(t))$ equals the rank of the considered system, then the particular system states are observable (locally weak observable). The system is globally observable if the local weak observability is proven for all points (excluding singular points) on manifold $\varvec{\mathcal {M}}$^8,56,59,65^. The potential singularities provide the problem, which makes this condition only sufficient, not necessary for all considered cases. It is necessary to check, how the parameters values, initial conditions, and inputs affect the singularity of $\partial _{\mathrm {\varvec{\nu }}} \varvec{\Phi }(\varvec{\nu }(t))$. In other words, the observability can be guaranteed in some time intervals contained in ${\mathbb {T}}$, in which duration is strongly dependent on the after-mentioned factors. If the system is unobservable (not all system states are observable) one can decompose its dynamics into two sub-dynamics, the former of which consists of observable states and the latter includes the stable unobservable part of the state vector. To make state decomposition properly, two assumptions must be met: (i) the observability (Jacobi) matrix must be invertible, and (2) unobservable states must be Lyapunov stable. These assumptions have important implications for further synthesis of the state observer, as shown in^78–83^.

Establishing the observability/detectability of a system using the classical approach outlined above does not guarantee (asymptotic) system state reconstruction in the face of uncertainty. Its presence imposes additional conditions closely related to the way it is modelled, and consequently to the synthesis of a state observer of a given type. As it has been mentioned, in the systems considered in this paper, the idea of unknown input sliding mode observers works well when eliminating the uncertainty leading to the appearance of unknown inputs^40,70–72,84,85^. However, the estimation of the system state using these observers requires that, besides the observability/detectability of the system, the matching condition is met^86^. For the system $\Sigma _\mathrm{d}$ under consideration, this condition is as follows:

7 $$\begin{aligned} L_{\rho _1}h \ne 0, \end{aligned}$$

what means that an element of field $\varvec{\rho }$ - $\rho _1$ is invariant with respect to the first component of the observability map (4), and that the $\text {span} \left\{ \varvec{\rho } \right\}$ does not annihilate $d\mathcal {O}$. Hence, the matching condition (7) states that the relative degree of system measured output *y*(*t*) with respect to $\Delta (\varvec{\nu }(t),t)$ (Lie differentiation with respect to $\varvec{\rho }(\varvec{\nu }(t))$) is equal to one. That means that there is a significant relation between the system measured output and the uncertainty, which makes a given (SMO) state observer to be able to cope with uncertain system dynamics via direct ’counter action’ imposed by a particular part of the correction term^40^.

However, the particular analysis for high dimensional or highly non-linear dynamics is well-known to be very troublesome. Moreover, this general methodology has not been ’globally’ derived for systems with unknown inputs. Therefore, the above classical approach is not always convenient, due to specific properties of a given system , e.g.,^44,48,60,61,87–90^. In order to overcome this problem, an alternative method based on the method of indistinguishable dynamics (indistinguishable state trajectories) can be used. According to this method, two states of a (non-linear) system with certain dynamics are said to be indistinguishable if the state trajectories associated with certain initial conditions are different, although the exogenous inputs and system measured outputs are the same^56,59,62,63^. Let us consider the system $\Sigma _{\mathrm{d}}$ with known input $\varvec{u}(t)$ and unknown inputs $\Delta (\varvec{\nu }(t),t)$ and $\Delta ^{'}(\varvec{\nu }(t),t)$^12,32,44,48,56,59–61,65,88^.

1. Two initial states $\varvec{\nu }_{0} \ne \varvec{\nu }_{0}^{'} \in \varvec{\mathcal {M}}$ are strongly u-indistinguishable, if for any $\varvec{u}(t)$ and for any pair of $\Delta (\varvec{\nu }(t),t), ~ \Delta ^{'}(\varvec{\nu }(t),t) \in {\mathbb {W}}$ (two unknown inputs not necessarily different from each other) hold: $y(\varvec{\nu }(t,\varvec{\nu }_{\mathrm{0}},\varvec{u}(t),\Delta (\varvec{\nu }(t),t))) = y(\varvec{\nu }(t,\varvec{\nu }_{\mathrm{0}}^{'},\varvec{u}(t),\Delta ^{'}(\varvec{\nu }(t),t)))$ and $\varvec{\nu }(t,\varvec{\nu }_{\mathrm{0}},\varvec{u}(t),\Delta (\varvec{\nu }(t),t)) \ne \varvec{\nu }(t,\varvec{\nu }_{\mathrm{0}}^{'},\varvec{u}(t),\Delta ^{'}(\varvec{\nu }(t),t))$. The set of strongly u-indistinguishable states from $\varvec{\nu }_{0}$ is denoted by $\mathcal {I}^{\mathrm{UI}}_{(\varvec{u}(t),\varvec{\nu }_{0})}$. The prefix ’u–($\cdot$)’ refers to the situation when the known input does not affect the observability property of the system. In the literature, this phenomenon is called the uniform observability.
2. System $\Sigma _{\mathrm{d}}$ is strongly u-observable if $\forall t \in {\mathbb {T}}$ for every $\varvec{\nu }_{0} \in \varvec{\mathcal {M}}$, and for any pair of $\Delta (\varvec{\nu }(t),t), ~ \Delta ^{'}(\varvec{\nu }(t),t) \in {\mathbb {W}}$ and for any $\varvec{u}(t)$ holds: $\mathcal {I}^{\mathrm{UI}}_{(\varvec{u}(t),\varvec{\nu }_{0})} = \{ \varvec{\nu }_{0} \}$. It means that the state trajectory is strongly u-distinguishable. In other words, the observability means that if (as assumed) $\varvec{\nu }_{0}^{'}$ is indistinguishable from $\varvec{\nu }_{0}$, and $y(\varvec{\nu }(t,\varvec{\nu }_{\mathrm{0}},\varvec{u}(t),\Delta (\varvec{\nu }(t),t))) \ne y(\varvec{\nu }(t,\varvec{\nu }_{\mathrm{0}}^{'},\varvec{u}(t),\Delta ^{'}(\varvec{\nu }(t),t)))$ and $\varvec{\nu }(t,\varvec{\nu }_{\mathrm{0}},\varvec{u}(t),\Delta (\varvec{\nu }(t),t)) \ne \varvec{\nu }(t,\varvec{\nu }_{\mathrm{0}}^{'},\varvec{u}(t),\Delta ^{'}(\varvec{\nu }(t),t))$ holds, then $\varvec{\nu }_{0}^{'} = \varvec{\nu }_{0}$ (the assumption on indistinguishability was a contradiction). Thus, the observable system state is only indistinguishable from ’itself’. It is worth emphasising that if system $\Sigma _{\mathrm{d}}$ is not associated with any indistinguishable initial conditions (trajectories), then it is fully observable.
3. System $\Sigma _{\mathrm{d}}$ is strongly u-detectable if $\forall t \in {\mathbb {T}}$ and for every $\varvec{\nu }_{0} \in \varvec{\mathcal {M}}$ for every $\varvec{\nu }^{'}_{0} \in \mathcal {I}^{\mathrm{UI}}_{(\varvec{u}(t),\varvec{\nu }_{0})}$, and for any $\varvec{u}(t)$ and also for any pair of $\Delta (\varvec{\nu }(t),t), ~\Delta ^{'}(\varvec{\nu }(t),t) \in {\mathbb {W}}$ that causes indistinguishable $\varvec{\nu }^{'}_{0}$. That means that $\forall t \in {\mathbb {T}} ~ y(\varvec{\nu }(t,\varvec{\nu }_{\mathrm{0}},\varvec{u}(t),\Delta (\varvec{\nu }(t),t))) = y(\varvec{\nu }(t,\varvec{\nu }_{\mathrm{0}}^{'},\varvec{u}(t),\Delta ^{'}(\varvec{\nu }(t),t)))$; from which it follows that $\varvec{\nu }(t,\varvec{\nu }_{\mathrm{0}},\varvec{u}(t),\Delta (\varvec{\nu }(t),t)) \rightarrow \varvec{\nu }(t,\varvec{\nu }_{\mathrm{0}}^{'},\varvec{u}(t),\Delta ^{'}(\varvec{\nu }(t),t))$ asymptotically.
4. System $\Sigma _{\mathrm{d}}$ is strongly observable (detectable) if it is strongly u-observable (u-detectable) for every $\varvec{u}(t)$.

The ’practical’ usage of the above terms is based on comparison of two different state trajectories generated for distinct initial conditions and unknown inputs $\forall t \in {\mathbb {T}}$. The first one is generated by the ’original’ (dependent on $\varvec{\nu }(t)$) system $\Sigma _{\mathrm{d}}$, and the other is generated by a ’copy’ (dependent on $\varvec{z}(t)$) of system $\Sigma _{\mathrm{d}}$. The dynamic meaning of this idea implies considering an extended model of the system which will consist of ’original’ and ’copied’ dynamics. Thus, it is necessary to derive the following ’error system’ (differential-algebraic equation - DAE) $\varvec{\varepsilon }(t) \in \Xi \subset {\mathbb {R}}^{n}$:

8 $$\begin{aligned} \varvec{\varepsilon }(t) \triangleq \varvec{\nu }(t) - \varvec{z}(t). \end{aligned}$$

Assuming that the initial conditions of both systems are different, i.e., $\varvec{\nu }_{0} \ne \varvec{z}_{0}$, and that the measured output and known inputs of both systems are the same, and also that different unknown inputs affect the system’s dynamics in the time interval ${\mathbb {T}}$:

- the observability means that the only possible state trajectories of the ’original’ and ’copied’ systems under these conditions are always equal (the ’error’ is zero) $\forall t \in {\mathbb {T}}$,
- the detectability means that the after-mentioned state trajectories mutually tend to each other asymptotically.

Several features of this approach are worth highlighting^12,60,61,87,88^:

1. The Lyapunov’ function-based approach may be used for analysing properties of system $\Sigma _{\mathrm{d}}$ such as observability and detectability.
2. Both observability and, especially, detectability are provable in the presence of unknown inputs.
3. The interpretation of the results obtained is relatively simple and based on classical terms linked with the theory of differential equations.
4. It can be used to characterise particular interactions between system states and exogenous inputs which involve ensuring observability/detectability.
5. By characterising zeros dynamics (known from the differential geometry based control approach, e.g.,^8^), it can be used to investigate observable and unobservable parts of the system.
6. Topological interpretation of sets of system states which make the system indistinguishable is possible via analysing properties of algebraic parts of the ’error system’ .
7. The results can be directly interpreted as global without invoking the after-mentioned concepts of local, weak or local weak observability.

### ’Bad inputs’ problem

As mentioned above, proving the observability/detectability of a non-linear system under the uncertainty resulting in the appearance of unknown inputs is a non-trivial task. Moreover, these unknown inputs may cause the model of the system under consideration to become unobservable/undetectable. The inputs that lead to this situation are usually called ’bad inputs’. Hence, the observability/detectability properties for all ’bad inputs’ are, in general, necessary to consider when designing a state UIO expected to provide good performance of the obtained estimates. It should be emphasised that the observability conditions must be strictly linked with a priori guarantee that the unknown inputs do not belong to ’bad inputs’^31,32,47,59,70,73,74,85,91^. For example, meeting the matching condition (7) ensures the above, and thus the designed unknown input sliding mode observer^40,70–72,84,85^ is able to correctly reconstruct the state vector.

Let us consider the system $\Sigma _{\mathrm{d}}$ again. If for any initial condition $\varvec{\nu }_{0}^{'}$ and for any unknown input $\Delta ^{'}(\varvec{\nu }(t),t)$ where $(\varvec{\nu }_{0},\Delta (\varvec{\nu }(t),t)) \ne (\varvec{\nu }_{0}^{'},\Delta ^{'}(\varvec{\nu }(t),t))$ holds $\forall t \in {\mathbb {T}}$ $y(\varvec{\nu }(t,\varvec{\nu }_{\mathrm{0}},\varvec{u},\Delta (\varvec{\nu }(t),t))) = y(\varvec{\nu }(t,\varvec{\nu }_{\mathrm{0}}^{'},\varvec{u}(t),\Delta ^{'}(\varvec{\nu }(t),t)))$, then the pair $(\varvec{\nu }_{0}^{'},\Delta ^{'}(\varvec{\nu }(t),t))$ is UI u-indistinguishable from the pair $(\varvec{\nu }_{0},\Delta (\varvec{\nu }(t),t))$. When the indistinguishable pairs do not exist, the system is UI u-observable (or UI u-distinguishable). Whereas if the indistinguishable trajectories converge asymptotically to each other, the system is UI u-detectable. That is, if $\forall t \in {\mathbb {T}}$ $y(\varvec{\nu }(t,\varvec{\nu }_{\mathrm{0}},\varvec{u}(t),\Delta (\varvec{\nu }(t),t))) = y(\varvec{\nu }(t,\varvec{\nu }_{\mathrm{0}}^{'},\varvec{u}(t),\Delta ^{'}(\varvec{\nu }(t),t)))$ that implies that $\varvec{\nu }(t,\varvec{\nu }_{\mathrm{0}},\varvec{u}(t),\Delta (\varvec{\nu }(t),t)) \rightarrow \varvec{\nu }(t,\varvec{\nu }_{\mathrm{0}}^{'},\varvec{u}(t),\Delta ^{'}(\varvec{\nu }(t),t))$ and, what is the most important, $\Delta ^{'}(\varvec{\nu }(t),t) \rightarrow \Delta (\varvec{\nu }(t),t)$ for $t \rightarrow \infty$.

Thus, the equivalence of meeting the matching condition (7) and UI observability/detectability is justified, which means that the conditions for the existence of the unknown input sliding mode observer are formulated. It is worth adding that when the matching condition is not met, then the uncertainty may not only strongly affect the performance of the obtained estimates, but also cause the unobservability of particular state variables. Several additional remarks referring to UI observability/detectability and the equivalence of two methods^61,92^ used to verify the observability/detectability property under uncertainty are given in Appendix B.

**Remark 1.** In general, the idea of ’bad inputs’ has been primarily developed in the context of the impact of particular known (control) inputs, which in some circumstances may lead to state unobservability^32,65,89,90^. If a particular control input distinguishes two different initial conditions $\varvec{\nu }_0$ and $\varvec{\nu }_0^{'}$, then it is called the universal input. Hence, its action on system dynamics does not induce the unobservability of state trajectories. However, this concept can be easily extended to the mentioned UI. Therefore , to avoid ambiguity, in this paper ’bad inputs’ concern only unknown inputs.

## Problem statement

In general, this paper is focused on the investigation of observability/detectability properties under uncertainty for a certain sub-class of the general class of affine non-linear dynamic systems. This sub-class is represented by a bioreactor in a wastewater treatment plant. The considered bioreactor model is based on a CSTR with the microbial growth reaction and microbial mortality with the aggregated substrate and biomass concentrations in aerobic phase. This model is considered the feasibility study for showing the important issues which can be encountered in the biochemical processes. The CSTR model of biochemical processes is less complex than the ASM model, but unfortunately, there is uncertainty in it. The main source of uncertainty in the model dynamics is the reaction kinetics function. In this paper, the reaction kinetics function is assumed to be the only uncertain part of the model under consideration. The concept of simplifying/transforming the reaction kinetics function in order to establish a model with exactly known dynamic part for analysing system properties and for further synthesis of control or estimation algorithms is widely known and gradually exploited. Many methods and approaches have been developed under distinct assumptions associated with the behaviour of system’s dynamics, e.g.,^3,7,37,81,93–97^. Modifications in the reaction kinetics function have been realised by taking into account various dependencies, e.g., time scales of the dynamics of state variables, available measurements, complexity of the reaction kinetics function, and predictive capabilities or uncertainties in the model dynamics. The idea of transforming the reaction kinetics function proposed in this paper is based on these experiences. However, the consequence of this approach is the appearance of unknown inputs. For a model of biochemical processes, in which the reaction kinetics function is an uncertain part of dynamics, proofing the observability/detectability properties is non-trivial. In addition, when analysing the observability/detectability together with the issue of unknown inputs, appropriate selection of system measured outputs must be made. This selection is essential for the synthesis of an observer that will enable system state reconstruction. To summarise, a systematic approach to analysing the observability/detectability of the non-linear uncertain bioreactor CSTR model with considering a given selection of system measured outputs is devised.

## Cognitive CSTR model

A CSTR with the microbial growth reaction and microbial mortality with the aggregated substrate $S(t) ~\left[ g/L\right]$, biomass $X(t) ~\left[ g/L\right]$ and dissolved oxygen $O(t) ~\left[ g/L\right]$ concentrations is one of the most widely used models of both the aerobic bioreactor and the sequencing batch reactor (SBR) in the aerobic phase^4,6,90,97–99^:

9 $$\begin{aligned} S(t) + O(t) \rightarrow X(t). \end{aligned}$$

For further considerations, $\forall t \in {\mathbb {T}}$ the set of all possible system states is defined as follows:

10 $$\begin{aligned} \varvec{\Omega } = \left\{ (X(t),S(t),O(t)) \in {\mathbb {R}}_{+}^3 :{\overline{X}} \ge X(t) \ge {\underline{X}}, ~ {\overline{S}} \ge S(t) \ge {\underline{S}}, ~ {\overline{O}} \ge O(t) \ge {\underline{O}} \right\} , \end{aligned}$$

where ${\overline{X}}$, ${\overline{S}}$, ${\overline{O}}$ and ${\underline{X}}$, ${\underline{S}}$, ${\underline{O}}$ are the real positive the upper and lower bounds of a particular variable, respectively. It is worth noting that $\varvec{\Omega }$ defined in this way is an invariant set meeting the condition of the general theory of dynamics of biochemical processes : $\varvec{\Omega } \subset \varvec{\mathcal {M}}\subset {\mathbb {R}}^3$^2,4,7,21,29^.

The cognitive CSTR model of the considered biochemical system includes the following phenomena^2,4,6,27,98–100^:

- microbial growth in the CSTR, described by the reaction kinetics function $r(t) ~\left[ g/h L\right]$,
- inflow to the CSTR and its outflow, described by the positive dilution rate $D(t) ~\left[ h^{-1}\right]$ $\forall t \in {\mathbb {T}}$, which is upper bounded by ${\overline{D}}$,
- gas-liquid transfer of dissolved oxygen to the CSTR, described by the positive term including the oxygen mass transfer coefficient ${k_{\mathrm{L}}a(t)} ~\left[ h^{-1}\right]$ $\forall t \in {\mathbb {T}}$, which is upper bounded by $\overline{k_{\mathrm{L}}a}$,
- microbial mortality, which is considered in the CSTR dynamics as follows:
  $$\begin{aligned} \begin{aligned} \text {biomass death:} ~ X(t)&\rightarrow X_\mathrm{d}(t),\\ \text {substrate and dissolved oxygen maintenance:} ~ O(t) + S(t) + X(t)&\rightarrow X(t), \end{aligned} \end{aligned}$$

where $X_\mathrm{d} ~\left[ g/L\right]$ is the dead biomass concentration modelled as a dissipating linear component in all equations of the model.

From the constituent mass balance law, the cognitive CSTR model taking into account the above-mentioned phenomena can be written as follows^2,4^:

11 $$\begin{aligned} \Sigma _\mathrm{CSTR}: {\left\{ \begin{array}{ll} {\dot{X}}(t) &{}= r(t) - \beta _\mathrm{m} X(t) - X(t)D(t)\\ {\dot{S}}(t) &{}= -\dfrac{1}{Y_{\mathrm{s}}}r(t) - m_\mathrm{s}X(t) + (S_\mathrm{in} - S(t))D(t)\\ {\dot{O}}(t) &{}= -\dfrac{1}{Y_{\mathrm{o}}}r(t) - m_\mathrm{o}X(t) + (O_\mathrm{in} - O(t))D(t) \\ {} &{}+ (O_\mathrm{s} - O(t)){k_{\mathrm{L}}a}(t)\\ X\left( t_0 \right) &{}= X_\mathrm{0}\\ S\left( t_0 \right) &{}= S_\mathrm{0}\\ O\left( t_0 \right) &{}= O_\mathrm{0} \end{array}\right. }, \end{aligned}$$

where: $\forall t \in {\mathbb {T}} :~ Y_{\mathrm{s}} ~\left[ -\right] \, Y_{\mathrm{o}} ~\left[ - \right]$ denote the positive yield coefficients of the growth of substrate and dissolved oxygen concentrations, respectively; $\forall t \in {\mathbb {T}} :~ S_{\mathrm{in}}> 0 ~\left[ g/L\right] , \, O_{\mathrm{in}} > 0 ~\left[ g/L\right]$ signify the concentrations of substrate and dissolved oxygen in the inflow to CSTR, respectively; $\forall t \in {\mathbb {T}} :~ O_{\mathrm{s}} ~\left[ g/L\right]$ stands for the positive saturation constant of dissolved oxygen concentration; $\forall t \in {\mathbb {T}} :~ \beta _{\mathrm{m}} > 0 ~\left[ h^{-1}\right]$ is the biomass mortality rate; $\forall t \in {\mathbb {T}} :~ m_{\mathrm{s}} ~\left[ h^{-1}\right] , \, m_{\mathrm{o}}~\left[ h^{-1}\right]$ denote the positive maintenance coefficients of the substrate and dissolved oxygen concentrations, respectively.

In model (11), the dilution rate (*D*(*t*)) and the gas-liquid transfer of dissolved oxygen (${k_{\mathrm{L}}a(t)}$) signify the bounded known inputs. In turn, the reaction kinetics function *r*(*t*) is one of the most crucial parts of the dynamics of biochemical processes. This is because it is a time-varying component that is a major source of uncertainty. A detailed explanation of selected aspects of the reaction kinetics function can be found in many literature references, e.g.,^1,2,4,21,22,26,29,40^. Taking this into account, in this paper all considered reaction kinetics functions are modelled as multiplication of a linear function related to *X*(*t*) and the Monod function related to *S*(*t*) or/and *O*(*t*). Therefore, the general form of the reaction kinetics function yields:

12 $$\begin{aligned} r(t) = \mu _{(\cdot )}(t)X(t), \end{aligned}$$

where $\forall t \in {\mathbb {T}} :~ \mu _{(\cdot )}(t)$ signifies the growth rate function which separately represents the effect of each component of the rate, and the lower index refers to the concentration which occurs in a given model.

In general, the Monod function is based on the Michaelis-Menten law. This function for *S*(*t*) or *O*(*t*) is given by:

13 $$\begin{aligned} \begin{aligned} \mu _{\mathrm{S}}(t) = \mu _{\mathrm{max}}(t)\dfrac{S(t)}{K_\mathrm{s}(t) + S(t)},\\ \mu _{\mathrm{O}}(t) = \mu _{\mathrm{max}}(t)\dfrac{O(t)}{K_\mathrm{o}(t) + O(t)}, \end{aligned} \end{aligned}$$

where $\forall t \in {\mathbb {T}} :~ \mu _{\mathrm{max}}(t) \in {\mathbb {R}}_{+} ~\left[ h^{-1}\right] , \, K_\mathrm{s}(t) \in {\mathbb {R}}_{+} ~\left[ g/L\right] , \, K_\mathrm{o}(t) \in {\mathbb {R}}_{+} ~\left[ g/L\right]$ are the time-varying coefficients of maximum specific growth rate, saturation of substrate concentration, and saturation of dissolved oxygen concentration, respectively.

Whereas for *S*(*t*) and *O*(*t*) is as follows:

14 $$\begin{aligned} \mu _{\text{SO}}(t) = \mu _\text{max}(t)\dfrac{S(t)}{K_\text{s}(t) + S(t)} \dfrac{O(t)}{K_\text{o}(t) + O(t)}. \end{aligned}$$

## CSTR model for observability/detectability analysis purposes

According to the discussion so far, the model of bioreactor for the proposed observability/detectability analysis should be of a general form (3). Therefore, the cognitive CSTR model (11) should be transformed. The following state variables, (known) model inputs (see Section "Cognitive CSTR model"), and unknown inputs are defined as:

15 $$\begin{aligned} \begin{aligned} \nu _1(t) \triangleq X(t), ~ \nu _2(t) \triangleq S(t), ~ \nu _3(t) \triangleq O(t), ~ u_1(t) \triangleq D(t), \\ u_2(t) \triangleq K_{\text{LA}}(t), ~ \Delta (\varvec{\nu }(t),t) \triangleq \left[ \mu _{\mathrm{SO}}(t) - \hat{\mu }_{(\cdot )}(t) \right] \nu _1(t), \end{aligned} \end{aligned}$$

where $\hat{\mu }_{(\cdot )}(t)$ denotes the exactly known substitute growth rate function.

Taking into account the general form (3) and the definition (15), the particular components of the CSTR model ($\Sigma _{\mathrm{CSTR}}^{\mathrm{est}}$) for the observability/detectability analysis is as follows :

16 $$\begin{aligned} \begin{aligned} \hat{\varvec{\varphi }}(\varvec{\nu }(t)) = \begin{bmatrix} \hat{\mu }_{(\cdot )}(t)\nu _1(t) - \beta _\mathrm{m} \nu _1(t) \\ -\dfrac{1}{Y_{\mathrm{s}}}\hat{\mu }_{(\cdot )}(t)\nu _1(t) - m_\mathrm{s}\nu _1(t) \\ -\dfrac{1}{Y_{\mathrm{o}}}\hat{\mu }_{(\cdot )}(t)\nu _1(t) - m_\mathrm{o}\nu _1(t) \end{bmatrix}, ~ \varvec{\vartheta }_{1}(\varvec{\nu }(t)) = \begin{bmatrix} -\nu _1(t) \\ S_{\mathrm{in}} - \nu _2(t) \\ O_{\mathrm{in}} - \nu _3(t) \end{bmatrix}, \\ \varvec{\vartheta }_{2}(\varvec{\nu }(t)) = \begin{bmatrix} 0 \\ 0 \\ O_{\mathrm{s}} - \nu _3(t) \end{bmatrix}, ~ \varvec{\rho }(\varvec{\nu }(t)) = \begin{bmatrix} 1 \\ -\dfrac{1}{Y_{\mathrm{s}}} \\ -\dfrac{1}{Y_{\mathrm{o}}} \end{bmatrix}. \end{aligned} \end{aligned}$$

The components $\hat{\varvec{\varphi }}(\varvec{\nu }(t))$, $\varvec{\vartheta }_{1}(\varvec{\nu }(t))$, $\varvec{\vartheta }_{2}(\varvec{\nu }(t))$, and $\varvec{\rho }(\varvec{\nu }(t))$ in (16) reveal the following general properties in terms of biochemical system dynamics:

1. Component $\hat{\varvec{\varphi }}(\varvec{\nu }(t))$, which is a substitution part of internal dynamics is strictly linked with the simplification of the reaction kinetics function. Many approaches to the modelling of reaction kinetics which lead to the determination of a given reaction kinetics function can be found in the literature. Some of them are based on neglecting the impact of slowly time-varying state variables either by treating them as constant parameters or excluding them due to their insignificant influence on process dynamics^5,7,37,95–97,101^. Other authors use a linear approximation of Monod, Haldane, etc. functions^3,5,37,82,83,93,97^. There are also methods based on linearisation^3,102^. A very rich survey of derived reaction kinetics functions can be found in^2^. As it has been shown, e.g., in^4,6,26^, the most common reaction kinetics function is the Monod function, which is also used in this paper (see (13)). It is worth adding that the structure of this function provides meeting the observability condition (injectivity of coordinate transformation). Therefore, it might be claimed that the Monod function is a proper candidate for being a substitute model of real reaction kinetics^40^. Once the structure of the reaction kinetics function has been defined in terms of a Monod function, it needs to be parameterised. The uncertain nature of reaction kinetics needs to consider parameters of this function as time-varying, which is included in the cognitive CSTR model (11). However, due to meeting the mentioned conditions, an approximating function $\hat{\mu }_{(\cdot )}(t)$ cannot have these kinds of parameters. Therefore, the original parameters of the reaction kinetics function are replaced by their time-invariant alternatives, which is done by calculating their mean values. To sum up, in this paper the exactly known substitute growth rate function $\hat{\mu }_{(\cdot )}(t)$ is based on the following two postulates:
  the structure of the substitute growth rate function is understood as a product of Monod functions,
  the parameters of the substitute growth rate function are assumed constant.
  Taking into account the above and (13), (14), the following forms of $\hat{\mu }_{(\cdot )}(t)$ are further considered:
  time-invariant Monod function related to *S*(*t*):
    17 $$\begin{aligned} \hat{\mu }_{\mathrm{S}}(t) = \mu _{\mathrm{max}}^0\dfrac{\nu _2(t)}{K_\mathrm{s}^0 + \nu _2(t)}, \end{aligned}$$
  time-invariant Monod function related to *O*(*t*):
    18 $$\begin{aligned} \hat{\mu }_{\mathrm{O}}(t) = \mu _{\mathrm{max}}^0\dfrac{\nu _3(t)}{K_\mathrm{o}^0 + \nu _3(t)}, \end{aligned}$$
  time-invariant product of two Monod functions related to *S*(*t*) and *O*(*t*):
    19 $$\begin{aligned} \hat{\mu }_{\mathrm{SO}}(t) = \mu _{\mathrm{max}}^0\dfrac{\nu _2(t)}{K_\mathrm{s}^0 + \nu _2(t)}\dfrac{\nu _3(t)}{K_\mathrm{o}^0 + \nu _3(t)}. \end{aligned}$$
  Time-invariant parameters $\mu _{\mathrm{max}}^0 \in {\mathbb {R}}_{+} ~\left[ h^\mathrm {-1}\right]$, $K_\mathrm{o}^0 \in {\mathbb {R}}_{+} ~\left[ g/L\right]$ and $K_\mathrm{s}^0 \in {\mathbb {R}}_{+} ~\left[ g/L\right]$ in (17)–(19) related to their time-varying originals are calculated as the mean values derived by using the knowledge about bounds of particular originals^40^.
2. Components $\varvec{\vartheta }_{1}(\varvec{\nu }(t))$ and $\varvec{\vartheta }_{2}(\varvec{\nu }(t))$ are straightforward due to a general theory of ’building’ a form of non-linear affine systems. Because *D*(*t*) and ${k_{\mathrm{L}}a(t)}$ are considered as exogenous known inputs to the CTSR, $\varvec{\vartheta }_{1}(\varvec{\nu }(t))$ and $\varvec{\vartheta }_{2}(\varvec{\nu }(t))$ are defined as coefficients which multiply particular inputs to the model. Nevertheless, it should be noted that in many papers, e.g.,^4,26,27^, ${k_{\mathrm{L}}a(t)}$ is treated as a time-varying parameter. Also, the observability/detectability analysis of a system similar to that under present consideration has been performed in based on this assumption^89,90^. However, in^98–100^ the authors presented profound explanation that ${k_{\mathrm{L}}a(t)}$ should be treated as an input to the model. The work in this paper also includes this approach. The key reason for this is the need to derive the component $\hat{\varvec{\varphi }}(\varvec{\nu }(t))$ in as simple form as possible.
3. Component $\varvec{\rho }(\varvec{\nu }(t))$ is related to the task of deriving $\Delta (\varvec{\nu }(t),t)$ using formula (2). Because the unknown input is defined as a difference between two elements related to the reaction kinetics function, the elements of $\varvec{\rho }(\varvec{\nu }(t))$ are either constant with value equal to one, or they express the yield coefficients.

The last important issue from the point of view of proving the observability/detectability of the model of the considered system is the choice of system measured outputs. In the papers devoted to state estimation of biochemical processes , especially those modelled as CSTR, a single measured output—biomass concentration—is usually used^4,14,26,27^. In real implementation of the state observer this proposition is in general possible but not essentially convenient. It is because of hardware aspects of the measuring tasks. On the other hand, in^21,27,90^, the authors proposed that the concentrations of substrate or dissolved oxygen have to be the system measured outputs. This case is more realistic and some interesting model properties for state observer synthesis purposes may be observed. For instance, unlike biomass concentration, the measurements of dissolved oxygen concentration are commonly used in the control system of dissolved oxygen in CSTR^98–100^.

The next Section presents the results of studies of various combinations of available measurements—*S*(*t*), *X*(*t*), *O*(*t*) and $\hat{\mu }_{(\cdot )}(t)$ forms (17)–(19), which give a wide view on the observability/detectability of the model of the biochemical system under consideration. Based on these studies, cases for which it is possible to synthesise a state observer (primarily SMO) guaranteeing proper state reconstruction are indicated.

## The observability/detectability analysis of the CSTR model

The observability/detectability analysis has been performed for nine cases covering a wide range of possible combinations of the after-mentioned system measured outputs and forms of the growth rate function. It was needed to state that for all considered cases of system measured output and growth kinetics function choice, the unknown input meets the matching condition (7) (is in certain sense ’observable’). In other words, due to the form of $\varvec{\rho }(\varvec{\nu }(t))$ in (16), it is easy to state that $L_{\rho _{1}}h \ne 0$ in all cases of system measured output choice:

20 $$\begin{aligned} \begin{aligned} L_{\rho _{1}}h = 1 ~ \text {if} ~ h(\varvec{\nu }(t)) = \nu _1(t), \\ L_{\rho _{1}}h = -\dfrac{1}{Y_{\mathrm{s}}} ~ \text {if} ~ h(\varvec{\nu }(t)) = \nu _2(t), \\ L_{\rho _{1}}h = -\dfrac{1}{Y_{\mathrm{o}}} ~ \text {if} ~ h(\varvec{\nu }(t)) = \nu _3(t). \end{aligned} \end{aligned}$$

Thus, the field $\varvec{\rho }$ is invariant with respect to the first component of the ’observability map’ and the $\text {span} \left\{ \varvec{\rho } \right\}$ does not annihilate $d\mathcal {O}$.

It is necessary to mention here a special (emergency) situation that can occur in a bioreactor. It is the wash-out state during which biomass is washed out, i.e., $\exists t_{\mathrm{X}} \in {\mathbb {T}} :~ X \left( t_{\mathrm{X}} \right) = 0$. From the point of view of the observability/detectability analysis based on model (16), *X*(*t*) remains zero $\forall t \ge t_{\mathrm{X}}$. The evolution of the *O*(*t*) and *S*(*t*) dynamics becomes independent of the impact of the reaction kinetics function and therefore obtaining any information about other states becomes extremely complicated or even impossible (it relies on the choice of the system measured output and the substitute reaction kinetics function). Hence, since $X \left( t_{\mathrm{X}} \right) = 0$, all states of the system are not observable for all known (control) and unknown inputs, and it is impossible to reconstruct all of the system states^12,89,90^. Certainly the wash-out state is highly undesired for the physical requirements of the entire process. Thus, this state is not considered in the further part of the observability/detectability analysis.

### Analysis based on differential geometry tools

To show how a given choice of $\hat{\mu }_{(\cdot )}(t)$ and *y*(*t*) affects the observability matrix $\partial _{\mathrm {\varvec{\nu }}}\varvec{\Phi }(\varvec{\nu }(t))$ the following analysis is given. According to the assumption that in all cases only one state variable is considered as a system measured output, i.e., $h(\varvec{\nu }(t)) = \nu _{j}(t)$, where $j \in \{1,2,3\}$, it is obvious that:

21 $$\begin{aligned} \forall t \in {\mathbb {T}} :~ \dfrac{\partial h_j}{\partial \nu _{i}} = 0, ~ i \ne j, ~ i = \overline{1,3}. \end{aligned}$$

Therefore, in general, the sufficient condition for observability of the system in a particular case is dependent on values of $dL_{\hat{\varvec{\varphi }}} h$ and $dL_{\hat{\varvec{\varphi }}}^2 h$. Let us make the first case analysis.

1. $y(t) = \nu _3(t)$ and $\hat{\mu }_{(\cdot )}(t) = \hat{\mu }_{\mathrm{O}}(t)$ Due to the knowledge about the structure of $\hat{\mu }_{\mathrm{O}}(t)$ it is easy to show that $\forall t \in {\mathbb {T}} :~ \hat{\varvec{\varphi }}\left( \nu _1(t),\nu _2(t),\nu _3(t)\right) \rightarrow \hat{\varvec{\varphi }}\left( \nu _1(t),\nu _3(t)\right)$. Therefore, $\forall t \in {\mathbb {T}} :~ \partial _{\nu _2} L_{\hat{\varvec{\varphi }}} h = 0$ and $\partial _{\nu _2} L_{\hat{\varvec{\varphi }}}^2 h = 0$, which results in obtaining the following observability matrix:
  22 $$\begin{aligned} \partial _{\mathrm {\varvec{\nu }}}\varvec{\Phi }(\varvec{\nu }(t)) = \begin{bmatrix} \partial _{\mathrm {\varvec{\nu }}} h \\ \partial _{\mathrm {\varvec{\nu }}} L_{\hat{\varvec{\varphi }}}h \\ \partial _{\mathrm {\varvec{\nu }}}L_{\hat{\varvec{\varphi }}}^2 h \end{bmatrix} = \begin{bmatrix} \partial _{\nu _1} h &{} \partial _{\nu _2} h &{} \partial _{\nu _3} h \\ \partial _{\nu _1} L_{\hat{\varvec{\varphi }}}h &{} \partial _{\nu _2} L_{\hat{\varvec{\varphi }}}h &{} \partial _{\nu _3} L_{\hat{\varvec{\varphi }}}h \\ \partial _{\nu _1}L_{\hat{\varvec{\varphi }}}^2 h &{} \partial _{\nu _2}L_{\hat{\varvec{\varphi }}}^2 h &{} \partial _{\nu _3}L_{\hat{\varvec{\varphi }}}^2 h \end{bmatrix} = \begin{bmatrix} 0 &{} 0 &{} 1 \\ \star &{} 0 &{} \star \\ \star &{} 0 &{} \star \end{bmatrix}, \end{aligned}$$
  where the symbol $\star$ denotes a certain non-linear expression calculated as particular partial derivative which is not equal to zero. The analytical calculations and explicit form of the observability matrix is not included in this paper. This is due to its bulkiness which the authors believe would not benefit the readability of the paper.
  The rank of the observability matrix (22) is not equal to the rank of the considered system model. Consequently, it is unobservable.
  However, it is worth noting that if we decompose the system dynamics into two parts, ’partial’ observability will be obtained. More specifically, the first part of the dynamics will meet the sufficient condition for observability if it consists of state variables $\nu _1(t)$ and $\nu _3(t)$. The second part, consisting of state variable $\nu _2(t)$, will be unobservable, which is due to the lack of this variable in $\hat{\mu }_{\mathrm{O}}(t)$. For decomposition purposes, Appendix A may be used. Then, the first part of the dynamics will be represented by subsystem ’$\varvec{x}(t)$’ containing $\nu _1(t)$ and $\nu _3(t)$, and by the second subsystem $\varvec{\xi }(t)$ containing $\nu _2(t)$ as parameter $\varvec{\xi }(t)$ in the unknown input $\Delta (\varvec{x}(t),\varvec{\xi }(t),t)$. A detailed procedure is shown in Appendix C.

An analogous analysis to case (1) was carried out for the other combinations and its results, together with case (1), are summarised in Table 1.

**Table 1:** Results of analysis based on differential geometry tools.

Case			Observability
No.	*y*(*t*)	$\hat{\mu }_{(\cdot )}(t)$	
(1)	$\nu _3(t)$	${\hat{\mu }_{\mathrm{O}}(t)}$	No
(2)	$\nu _1(t)$	${\hat{\mu }_{\mathrm{O}}(t)}$	No
(3)	$\nu _2(t)$	${\hat{\mu }_{\mathrm{O}}(t)}$	Yes
(4)	$\nu _3(t)$	$\hat{\mu }_{\mathrm{S}}(t)$	Yes
(5)	$\nu _1(t)$	${\hat{\mu }_{\mathrm{S}}(t)}$	No
(6)	$\nu _2(t)$	${\hat{\mu }_{\mathrm{S}}(t)}$	No
(7)	$\nu _1(t)$	${\hat{\mu }_{\mathrm{SO}}(t)}$	Yes
(8)	$\nu _2(t)$	${\hat{\mu }_{\mathrm{SO}}(t)}$	Yes
(9)	$\nu _3(t)$	${\hat{\mu }_{\mathrm{SO}}(t)}$	Yes

To complement the above conclusions, the following comments are added:

- In cases, (7), (8) and (9), the considered model meets the sufficient condition of observability, which is due to the effect of consistence of $\nu _2(t)$ and $\nu _3(t)$ ensured by the structure of the substitute growth rate function. Naturally, $\nu _1(t)$ is a part of all particular $\hat{\varvec{\varphi }}(\varvec{\nu }(t))$, therefore it is always an observable state variable. However, from the point of view of further SMO synthesis , formula (19) is complicated, and, as a result, the established state coordinate transformations (6) would contain very complex components. Therefore, when designing SMO, this formula is further simplified, but without losing the observability property.
- Cases (1), (2), (5) and (6) concern situations when the considered model is unobservable. However, it is possible to separate the observable part of the system dynamics. This translates into the observability of two of the three state variables. This situation is an effect of the possibility of decomposition of the considered model, as explained in Appendices A and C.
- Cases (3) and (4) are the most interesting. It is due to deriving the methodology of the observability analysis for further convenient synthesis of SMO. Both situations give certain combinations of substitute growth rate function and system measured outputs which guarantee the observability of all system states and consequently their reconstructability.

**Remark 2.** As it has been mentioned, the observability or unobservability of the particular state variable is strictly dependent on its occurrence in the considered function $\hat{\mu }_{(\cdot )}(t)$. Taking into account that SMO might be able to generate the system states estimates $\hat{\varvec{\nu }}(t)$ under uncertain reaction kinetics function it always follows to $\hat{\varvec{\nu }}(t) \rightarrow \varvec{\nu }(t)$ asymptotically when the system is observable.

### Analysis based on the method of indistinguishable state trajectories

To show how a given choice of $\hat{\mu }_{(\cdot )}(t)$ and *y*(*t*) affects the observability/detectability properties, the following analysis is given. The ’copied’ (dependent on $\varvec{z}(t)$) system $\Sigma _{\mathrm{CSTR}}^{\mathrm{est}}$ is structurally the same as the ’original’ (dependent on $\varvec{\nu }(t)$) one from (16). According to Section Observability/detectability analysis for non-linear systems, the ’error’ is defined as follows:

23 $$\begin{aligned} \varvec{\varepsilon }(t) \triangleq \varvec{\nu }(t) - \varvec{z}(t) ~ \rightarrow ~ \varvec{z}(t) = \varvec{\nu }(t) - \varvec{\varepsilon }(t). \end{aligned}$$

Moreover, the difference between uncertain parts of the dynamics of the ’original’ and ’copied’ systems is defined in the following way^60,61,87,88^:

24 $$\begin{aligned} \delta \Delta (\varvec{\nu }(t),\varvec{z}(t),t) \triangleq \Delta (\varvec{\nu }(t),t) - \Delta ^{'}(\varvec{z}(t),t), \end{aligned}$$

where $\Delta (\varvec{\nu }(t),t)$ and $\Delta ^{'}(\varvec{z}(t),t)$ are the unknown inputs considered in a particular ’original’ system and its ’copy’ defined in (15), respectively.

Symbol $\delta \Delta (\varvec{\nu }(t),\varvec{z}(t),t)$ is a universal symbol for denoting the variance (error) of the unknown input in all considered cases; henceforth, denoted as $\delta \Delta$ for simplicity. The initial conditions are not equal, i.e., $\varvec{\nu }\left( t_0 \right) \ne \varvec{z}\left( t_0 \right)$, which imposes a potential different behaviour of the integral curves^44,48,61,87,89,90^.

For all considered cases, the dynamics of the ’error system’ $\Sigma _{\mathrm{E}}^{(\cdot )}$ reads:

25 $$\begin{aligned} \Sigma _{\mathrm{E}}^{(\cdot )} :{\left\{ \begin{array}{ll} {\dot{\varepsilon }}_1(t) &{}= \hat{\mu }_{(\cdot )}(t)\nu _1(t) - \hat{\mu }_{(\cdot )}^{'}(t) \left[ \nu _1(t) - \varepsilon _1(t) \right] \\ {} &{}- \beta _{\mathrm{m}}\varepsilon _1(t) - u_1(t) \varepsilon _1(t) + \delta \Delta \\ {\dot{\varepsilon }}_2(t) &{}= -\dfrac{1}{Y_{\mathrm{s}}} \left[ \hat{\mu }_{(\cdot )}(t)\nu _1(t) - \hat{\mu }_{(\cdot )}^{'}(t) \left[ \nu _1(t) - \varepsilon _1(t) \right] + \delta \Delta \right] \\ &{}- m_{\mathrm{s}} \varepsilon _1(t) - u_1(t) \varepsilon _2(t) \\ {\dot{\varepsilon }}_3(t) &{}= -\dfrac{1}{Y_{\mathrm{o}}} \left[ \hat{\mu }_{(\cdot )}(t)\nu _1(t) - \hat{\mu }_{(\cdot )}^{'}(t) \left[ \nu _1(t) - \varepsilon _1(t) \right] + \delta \Delta \right] \\ &{}- m_{\mathrm{o}} \varepsilon _1(t) - \left[ u_1(t) + u_2(t) \right] \varepsilon _3(t) \\ \varvec{\varepsilon }\left( t_0 \right) &{}= \varvec{\varepsilon }_0 \end{array}\right. }. \end{aligned}$$

System (25) has an analogous form to the general form of the affine system invoked in (1), whereas its dynamics is a 6-dimensional manifold composed as the Cartesian product of the original state space and the ’error’ ($\varvec{\varepsilon }(t) \in \varvec{\Xi }$) space, i.e., $\left[ \varvec{\nu }^{\mathrm{T}}(t), ~ \varvec{\varepsilon }^{\mathrm{T}}(t) \right] ^{\mathrm{T}} \in \varvec{\Omega } \times \varvec{\Xi } = \varvec{\Psi } \subset {\mathbb {R}}_{+}^{3} \times {\mathbb {R}}^{3}$.

Due to the fact that system (25) presents a general form of ’error’ dynamics, the following comments are given:

- Dynamics (1) is not affected by (25); however, the inverse relation is necessary for the purposes of the observability/detectability analysis.
- System $\Sigma _{\mathrm{E}}^{(\cdot )}$ is the ’base dynamics’ for each case considered in the observability/detectability analysis.
- Symbol $\hat{\mu }_{(\cdot )}^{'}(t)$ is a universal symbol for denoting the Monod function for the ’copied’ system, the structure and parametrisation of which is the same as that of the ’original’ system.

The pair of systems $\Sigma _{\mathrm{CSTR}}^{\mathrm{est}}$ and $\Sigma _{\mathrm{E}}^{(\cdot )}$ has a so-called strongly indistinguishable dynamics, which means that the state trajectories of these systems are indistinguishable. By interpreting the dynamic behaviour of $\Sigma _{\mathrm{E}}^{(\cdot )}$ ’supplied’ by known and unknown inputs, measurements, and the state behaviour of (3), it may be stated that the original system is observable or not. Hence, by invoking to propositions from^44,48,87^ and the content of Section "The observability/detectability analysis of the CSTR model", the following extended definitions are given:

- System $\Sigma _{\mathrm{CSTR}}^{\mathrm{est}}$ is globally strongly u-observable (briefly: strongly u-observable) if and only if for any pair $\Delta (\cdot )$ and $\Delta ^{'}(\cdot )$, for every input-output system’s behaviour, and for every $\varvec{\varepsilon }\left( t_0 \right)$ there is no solution trajectory in ${\mathbb {T}}$ for $\Sigma _{\mathrm{E}}^{(\cdot )}$. There is only the trivial solution given by $\varvec{\varepsilon }(t) = \overline{\varvec{\varepsilon }} = 0 ~ \forall t \in {\mathbb {T}}$, where $\overline{\varvec{\varepsilon }}$ is an equilibrium point of error dynamics (so-called ’zero point’). The lack of trajectory solution means that there are no time-varying integral curves derived from ’error system’ dynamics which would coincide with the stability of ’zero point’, but there exists only a constant solution at this point indeed. In this situation, all state variables, as well as unknown inputs, are observable.
- System $\Sigma _{\mathrm{CSTR}}^{\mathrm{est}}$ is locally strong u-observable if and only if the conditions for global strongly u-observability hold only in a certain neighbourhood $\varvec{\mathcal {V}} \subset \varvec{\Xi }$ of ’zero point’.
- System $\Sigma _{\mathrm{CSTR}}^{\mathrm{est}}$ is globally strongly u-detectable (briefly: strongly u-detectable) if and only if for any pair $\Delta (\cdot )$ and $\Delta ^{'}(\cdot )$, for every input-output system’s behaviour, and for every $\varvec{\varepsilon }\left( t_0 \right) \ne \overline{\varvec{\varepsilon }}$, the ’zero point’ of $\Sigma _{\mathrm{E}}^{(\cdot )}$ is globally an attractive point for all trajectories defined in ${\mathbb {T}}$. It means that for any initial condition $\varvec{\varepsilon }\left( t_0 \right)$, the dynamics of ’error system’ $\varvec{\varepsilon }(t) \rightarrow 0$ when $t \rightarrow \infty$. In this situation, at least one state variable must be detectable and the unknown input must be observable or detectable.
- System $\Sigma _{\mathrm{CSTR}}^{\mathrm{est}}$ is locally strongly u-detectable if and only if the conditions for global strong u-detectability hold only in a certain neighbourhood $\varvec{\mathcal {V}} \subset \varvec{\Xi }$ of ’zero point’.

The understanding of local and global definitions of observability and detectability based on indistinguishable dynamics is not equivalent to the notions of local, weak and local weak observability introduced in Section "Observability/detectability analysis for non-linear systems". Whereas the abovementioned notions based on differential geometry tools invoke the properties of the manifold $\varvec{\mathcal {M}}$ and are associated with state-space transformations (precisely Lie derivative based co-distributions), the meaning of the concepts presented in this Section comply with classical understanding of the theory of stability. More specifically, they are natural extensions of definitions presented in Section "Observability/detectability analysis for non-linear systems". Local observability/detectability may be constituted due to the occurrence of the potential impact of ’bad inputs’ or the existence of singular points which can make the system unobservable/undetectable in some regions of $\varvec{\mathcal {M}}$. By using the Lyapunov stability^9^, it is possible to state that the dynamics of the ’error system’ is asymptotically stable for all initial states or it is stable only for their particular subset (local asymptotic stability). For the geometric approach, an analogous interpretation may be presented, e.g., if the rank of the observability matrix is equal to the system rank everywhere, then the system is globally observable the necessary and sufficient conditions for observability are met. If not, it might be claimed that the system is locally observable in some region of manifold $\varvec{\mathcal {M}}$. However, as it has been emphasised in Section "Observability/detectability analysis for non-linear systems" the classical approach has minimal aptitude to perform a detectability analysis.

Thus, if the inputs and measured outputs of both systems, i.e., $\Sigma _{\mathrm{CSTR}}^{\mathrm{est}}$ and $\Sigma _{\mathrm{E}}^{(\cdot )}$, are identical in ${\mathbb {T}}$, the observability property is equivalent to the statement that the only possible solution trajectories of the ’original’ and ’copied’ systems are always equal. It means that particular components of $\varvec{\varepsilon }(t)$ are equal to zero $\forall t \in {\mathbb {T}}$. Whereas, the detectability property is equivalent to the statement that the abovementioned trajectories mutually tend to each other asymptotically for $t \rightarrow \infty$. Naturally, that means that particular components of $\varvec{\varepsilon }(t)$ tend to zero $\forall t \in {\mathbb {T}}$.

The next Section analyses selected cases from Section "Analysis based on differential geometry tools". Finally, for comparative purposes, an analogous analysis is carried out in the absence of uncertainty in the considered model.

#### Analysis for the CSTR model with uncertain dynamics

**Case:** (1) - $$y(t) = \nu _3(t)$$ and $$\hat{\mu }_{(\cdot )}(t) = \hat{\mu }_{\mathrm{O}}(t)$$.

The system measured output is: $y(t) = \nu _3(t)$. It implies that $\forall t \in {\mathbb {T}} :~ \varepsilon _3(t) = 0$, and ${\dot{\varepsilon }}_3(t) = 0$. Thus, the third equation in (25) yields:

26 $$\begin{aligned} \begin{aligned} 0&= -\dfrac{1}{Y_{\mathrm{o}}} \left[ \hat{\mu }_{\mathrm{O}}(t)\nu _1(t) - \hat{\mu }_{\mathrm{O}}^{'}(t) \left[ \nu _1(t) - \varepsilon _1(t) \right] \right] - m_{\mathrm{o}}\varepsilon _1(t) -\dfrac{1}{Y_{\mathrm{o}}} \delta \Delta ~ \rightarrow \\ \delta \Delta&= -\left[ \hat{\mu }_{\mathrm{O}}(t)\nu _1(t) - \hat{\mu }_{\mathrm{O}}^{'}(t) \left[ \nu _1(t) - \varepsilon _1(t)\right] \right] - Y_{\mathrm{o}}m_{\mathrm{o}}\varepsilon _1(t). \end{aligned} \end{aligned}$$

Taking into account that $\varepsilon _3(t) = 0$, the growth rate functions of the ’original’ and ’copied’ systems are $\hat{\mu }_{\mathrm{O}}(t) = \hat{\mu }_{\mathrm{O}}^{'}(t)$ $\forall t \in {\mathbb {T}}$. Hence, (26) is rewritten as follows:

27 $$\begin{aligned} \delta \Delta = -\varepsilon _1(t)\left[ \hat{\mu }_{\mathrm{O}}(t) + Y_{\mathrm{o}}m_{\mathrm{o}} \right] . \end{aligned}$$

It is worth mentioning that due to the boundedness of $\nu _3(t)$, the growth rate function $\hat{\mu }_{\mathrm{O}}(t)$ is also bounded.

Considering the above, (25) is transformed to the following DAE system:

28 $$\begin{aligned} \Sigma _{\mathrm{E}}^{(1)} :{\left\{ \begin{array}{ll} {\dot{\varepsilon }}_1(t) &{}= -\varepsilon _1(t) \left[ \beta _{\mathrm{m}} + u_1(t) + Y_{\mathrm{o}}m_{\mathrm{o}} \right] \\ {\dot{\varepsilon }}_2(t) &{}= - \varepsilon _2(t)u_1(t) - \varepsilon _1(t) \left[ m_{\mathrm{s}} + \dfrac{1}{Y_{\mathrm{s}}}Y_{\mathrm{o}}m_{\mathrm{o}} \right] \\ \delta \Delta &{}= -\varepsilon _1(t)\left[ \hat{\mu }_{\mathrm{O}}(t) + Y_{\mathrm{o}}m_{\mathrm{o}} \right] \end{array}\right. }. \end{aligned}$$

The first differential equation of system $\Sigma _{\mathrm{E}}^{(1)}$ is globally asymptotically stable, so the error $\varepsilon _1(t) ~ \rightarrow ~ 0$. Therefore, the second differential equation of $\Sigma _{\mathrm{E}}^{(1)}$ can be written as follows:

29 $$\begin{aligned} {\dot{\varepsilon }}_2(t) = -u_1(t)\varepsilon _2(t) - \gamma _2(t)\left[ m_{\mathrm{s}} + \dfrac{1}{Y_{\mathrm{s}}}Y_{\mathrm{o}}m_{\mathrm{o}} \right] , \end{aligned}$$

where $\varepsilon _1(t) \equiv \gamma _2(t) = \varepsilon _1\left( t_0 \right) \exp \left( -\int _{t_0}^{t} u_1(\tau ) d\tau -\left[ \beta _{\mathrm{m}} + Y_{\mathrm{o}}m_{\mathrm{o}} \right] (t-t_0) \right) \rightarrow 0$, for $t \rightarrow \infty$ is the solution of the first differential equation ($\varepsilon _1(t)$ dependent) in (28).

The two known inputs of the system are always positive, therefore it may be stated that the solutions of differential equations in (28) and (29) tend to zero asymptotically for any initial condition. This leads to $\delta \Delta$ in (28) approaching zero if $\varepsilon _1(t) \rightarrow 0$ asymptotically. Therefore, in case (1), the state variables $\nu _1(t)$ and $\nu _2(t)$ are strongly u-detectable, whereas $\delta \Delta$ is detectable. The potential situation (but physically impossible due to permanent system activation) of zeroing both growth rate functions due to $\nu _3(t) = 0$ does not affect the conclusion.

**Case:** (5) - ($$y(t) = \nu _1(t)$$ and $$\hat{\mu }_{(\cdot )}(t) = \hat{\mu }_{\mathrm{S}}(t)$$).

The system measured output is: $y(t) = \nu _1(t)$. It implies that $\forall t \in {\mathbb {T}} :~ \varepsilon _1(t) = 0$, and ${\dot{\varepsilon }}_1(t) = 0$. Thus, the first equation in (25) yields:

30 $$\begin{aligned} 0 = \left[ \hat{\mu }_{\mathrm{S}}(t) - \hat{\mu }_{\mathrm{S}}^{'}(t)\right] \nu _1(t) + \delta \Delta ~ \rightarrow ~ \delta \Delta = \left[ \hat{\mu }_{\mathrm{S}}^{'}(t) - \hat{\mu }_{\mathrm{S}}(t)\right] \nu _1(t). \end{aligned}$$

Taking into account (30), and by performing proper substitutions, (25) is transformed to the following DAE system:

31 $$\begin{aligned} \Sigma _{\mathrm{E}}^{(5)} :{\left\{ \begin{array}{ll} {\dot{\varepsilon }}_2(t) &{}= - u_1(t) \varepsilon _2(t) \\ {\dot{\varepsilon }}_3(t) &{}= - \left[ u_1(t) + u_2(t) \right] \varepsilon _3(t) \\ \delta \Delta &{}= \left[ \hat{\mu }_{\mathrm{S}}^{'}(t) - \hat{\mu }_{\mathrm{S}}(t) \right] \nu _1(t) \\ \varvec{\varepsilon }\left( t_0 \right) &{}= \varvec{\varepsilon }_0 \end{array}\right. }. \end{aligned}$$

The two known inputs of the system are always positive, therefore it may be stated that the solutions of differential equations in (31) tend to zero asymptotically for any initial condition. Since both growth rate functions $\hat{\mu }_{\mathrm{S}}^{'}(t)$ and $\hat{\mu }_{\mathrm{S}}(t)$ are the Monod functions, if $\varepsilon _2(t) \rightarrow 0$ then $\hat{\mu }_{\mathrm{S}}^{'}(t) \rightarrow \hat{\mu }_{\mathrm{S}}(t)$ asymptotically without the occurrence of causing ’bad inputs’. Moreover, due to the boundedness of all state variables, the growth rate function, and consequently the reaction kinetics function, do not tend to infinity. Therefore, in case (5) the system state variables $\nu _2(t)$ and $\nu _3(t)$ are strongly u-detectable.

To show the convergence of the growth rate functions, by assuming that $\forall t \in {\mathbb {T}} :~ \nu _1(t) \ne 0$, the following calculations are performed:

32 $$\begin{aligned} \begin{aligned} \delta \Delta&= \mu _{\mathrm{max}}^0 \left[ \dfrac{\nu _2(t)}{K_\mathrm{s}^0 - \nu _2(t)} - \dfrac{\nu _2(t) - \varepsilon _2(t)}{K_\mathrm{s}^0 + \nu _2(t) - \varepsilon _2(t)}\right] \nu _1(t), \\ \delta \Delta&= \mu _{\mathrm{max}}^0 \left[ \dfrac{\left( K_\mathrm{s}^0 + \nu _2(t) \right) \left( \nu _2(t) - \varepsilon _2(t) \right) - \nu _2(t)\left( K_\mathrm{s}^0 + \nu _2(t) - \varepsilon _2(t) \right) }{\left( K_\mathrm{s}^0 + \nu _2(t) \right) \left( K_\mathrm{s}^0 + \nu _2(t) - \varepsilon _2(t) \right) } \right] \nu _1(t), \\ \delta \Delta&= \mu _{\mathrm{max}}^0 \left[ \dfrac{K_\mathrm{s}^0 \varepsilon _2(t)}{\left( K_\mathrm{s}^0 + \nu _2(t) \right) \left( K_\mathrm{s}^0 + \nu _2(t) - \varepsilon _2(t) \right) } \right] \nu _1(t). \end{aligned} \end{aligned}$$

Therefore, if $\varepsilon _2(t) \rightarrow 0$, then $\delta \Delta$ must also asymptotically tend to zero, which means that the uncertainty is detectable.

However, in this moment, the considerations about the observability/detectability of $\delta \Delta$ in (31) should be divided into two situations. The first situation takes place when the state variable $\nu _1(t)$ is always positive, whereas the second is linked with the wash-out state. In the second situation, the state variable $\nu _1(t)$ becomes zero either asymptotically (A) or due to initial condition (IC) $\nu _1\left( t_0 \right) = 0$, while in the first situation, where $\nu _1(t)$ is always positive (and bounded by assumption), the right-hand side of the third equation in (31) is not equal to zero until the growth rate functions become equal to each other. It leads to the conclusion that the uncertainty $\delta \Delta$ is approaching zero when $\varepsilon _2(t) \rightarrow 0$. That means that $\delta \Delta$ is detectable. The detectability of $\nu _2(t)$ and $\nu _3(t)$ is not affected by this assumption. In the second situation, when the initial condition of state variable $\nu _1(t)$ is equal to zero, the right-hand side of the third equation in (31) must always be equal to zero. This leads to the conclusion that the uncertainty $\delta \Delta$ is observable. In turn, if the state variable $\nu _1(t)$ tends to zero asymptotically, the uncertainty $\delta \Delta$ is detectable until $\nu _1(t) = 0$. This provides the observability of $\delta \Delta$. In both cases of the second situation, this property is independent of asymptotic approaching of the growth rate functions. Also, the detectability of $\nu _2(t)$ and $\nu _3(t)$ is not affected by this assumption.

An analogous analysis to cases (1) and (5) was carried out for the other combinations and its results, together with cases (1) and (5), are summarised in Table 2.

**Table 2:** Results of analysis based on indistinguishable dynamics with the uncertainty.

Case			Observability				Detectability			
No.	*y*(*t*)	$\hat{\mu }_{(\cdot )}(t)$	$\nu _1(t)$	$\nu _2(t)$	$\nu _3(t)$	$\delta \Delta$	$\nu _1(t)$	$\nu _2(t)$	$\nu _3(t)$	$\delta \Delta$
(1)	$\nu _3(t)$	$\hat{\mu }_{\mathrm{O}}(t)$	No	No	–	No	Yes	Yes	–	Yes
(2)	$\nu _1(t) > 0$	$\hat{\mu }_{\mathrm{O}}(t)$	–	No	No	No	–	Yes	Yes	Yes
	$\nu _1(t) = 0$ (IC)		–	No	No	Yes	–	Yes	Yes	No
	$\nu _1(t) = 0$ (A)		–	No	No	No	–	Yes	Yes	No
(3)	$\nu _2(t)$	$\hat{\mu }_{\mathrm{O}}(t)$	Yes	–	Yes	Yes	Yes	–	Yes	Yes
(4)	$\nu _3(t)$	$\hat{\mu }_{\mathrm{S}}(t)$	Yes	Yes	–	Yes	Yes	Yes	–	Yes
(5)	$\nu _1(t) > 0$	$\hat{\mu }_{\mathrm{S}}(t)$	–	No	No	No	-	Yes	Yes	Yes
	$\nu _1(t) = 0$ (IC)		–	No	No	Yes	–	Yes	Yes	Yes
	$\nu _1(t) = 0$ (A)		–	No	No	No	–	Yes	Yes	Yes
(6)	$\nu _2(t)$	$\hat{\mu }_{\mathrm{S}}(t)$	No	–	No	No	Yes	–	Yes	Yes
(7)	$\nu _1(t) > 0$	$\hat{\mu }_{\mathrm{SO}}(t)$	–	Yes	Yes	Yes	–	Yes	Yes	Yes
	$\nu _1(t) = 0$ (IC)		–	Yes	Yes	No	–	Yes	Yes	No
	$\nu _1(t) = 0$ (A)		–	Yes	Yes	No	-	Yes	Yes	No
(8)	$\nu _2(t)$	$\hat{\mu }_{\mathrm{SO}}(t)$	Yes	–	Yes	Yes	Yes	–	Yes	Yes
(9)	$\nu _3(t)$	$\hat{\mu }_{\mathrm{SO}}(t)$	Yes	Yes	–	Yes	Yes	Yes	–	Yes

#### Analysis for the CSTR model without uncertain dynamics

In this Section, for comparative purposes, an analogous analysis to that presented in Section “Analysis for the CSTR model with uncertain dynamics” is carried out in the absence of uncertainty in the model under consideration.

**Case:** (1) - $$y(t) = \nu _3(t)$$ and $$\hat{\mu }_{(\cdot )}(t) = \hat{\mu }_{\mathrm{O}}(t)$$.

The system measured output is: $y(t) = \nu _3(t)$. It implies that $\forall t \in {\mathbb {T}} :~ \varepsilon _3(t) = 0$, and ${\dot{\varepsilon }}_3(t) = 0$. Thus, the first equation in (25) yields:

33 $$\begin{aligned} 0 = -\left[ \hat{\mu }_{\mathrm{O}}(t)\nu _1(t) - \hat{\mu }_{\mathrm{O}}^{'}(t) \left[ \nu _1(t) - \varepsilon _1(t) \right] \right] - Y_{\mathrm{o}}m_{\mathrm{o}}\varepsilon _1(t). \end{aligned}$$

Taking into account that $\varepsilon _3(t) = 0$, the growth rate functions of the ’original’ and ’copied’ systems are $\hat{\mu }_{\mathrm{O}}(t) = \hat{\mu }_{\mathrm{O}}^{'}(t)$ $\forall t \in {\mathbb {T}}$. Hence, (33) is rewritten as follows:

34 $$\begin{aligned} 0 = -\varepsilon _1(t)\left[ \hat{\mu }_{\mathrm{O}}(t) + Y_{\mathrm{o}}m_{\mathrm{o}} \right] . \end{aligned}$$

It is worth mentioning that due to the boundedness of $\nu _3(t)$, the growth rate function $\hat{\mu }_{\mathrm{O}}(t)$ is also bounded.

Considering the above, (25) is transformed to the following DAE system:

35 $$\begin{aligned} \Sigma _{\mathrm{E}}^{(1)} :{\left\{ \begin{array}{ll} {\dot{\varepsilon }}_1(t) &{}= -\varepsilon _1(t) \left[ \beta _{\mathrm{m}} + u_1(t) + Y_{\mathrm{o}}m_{\mathrm{o}} \right] \\ {\dot{\varepsilon }}_2(t) &{}= - \varepsilon _2(t)u_1(t) - \varepsilon _1(t) \left[ m_{\mathrm{s}} + \dfrac{1}{Y_{\mathrm{s}}}Y_{\mathrm{o}}m_{\mathrm{o}} \right] \\ 0 &{}= -\varepsilon _1(t)\left[ \hat{\mu }_{\mathrm{O}}(t) + Y_{\mathrm{o}}m_{\mathrm{o}} \right] \end{array}\right. }. \end{aligned}$$

The third equation of system $\Sigma _{\mathrm{E}}^{(1)}$ is equal to zero only if $\varepsilon _1(t) = 0$ and ${\dot{\varepsilon }}_1(t) = 0$. This leads to the conclusion that the state variable $\nu _1(t)$ is strongly u-observable. Therefore, the second differential equation of $\Sigma _{\mathrm{E}}^{(1)}$ can be written as follows:

36 $$\begin{aligned} {\dot{\varepsilon }}_2(t) = -u_1(t)\varepsilon _2(t) - \gamma _4(t)\left[ m_{\mathrm{s}} + \dfrac{1}{Y_{\mathrm{s}}}Y_{\mathrm{o}}m_{\mathrm{o}} \right] , \end{aligned}$$

where $\varepsilon _1(t) \equiv \gamma _4(t) = \varepsilon _1\left( t_0 \right) \exp \left( -\int _{t_0}^{t} u_1(\tau ) d\tau -\left[ \beta _{\mathrm{m}} + Y_{\mathrm{o}}m_{\mathrm{o}} \right] (t-t_0) \right) \rightarrow 0$, for $t \rightarrow \infty$ is the solution of the first differential equation ($\varepsilon _1(t)$ dependent) in (35).

The two known inputs of the system are always positive, therefore it may be stated that the solutions of differential equations in (35) and (36) tend to zero asymptotically for any initial condition. Therefore, in case (1), the state variable $\nu _1(t)$ is strongly u-observable, whereas state variable $\nu _2(t)$ is strongly u-detectable. The potential situation (but physically impossible due to permanent system activation) of zeroing both growth rate functions due to $\nu _3(t) = 0$ does not affect the conclusion.

**Case:** (5) - ($$y(t) = \nu _1(t)$$ and $$\hat{\mu }_{(\cdot )}(t) = \hat{\mu }_{\mathrm{S}}(t)$$).

The system measured output is: $y(t) = \nu _1(t)$. It implies that $\forall t \in {\mathbb {T}} :~ \varepsilon _1(t) = 0$, and ${\dot{\varepsilon }}_1(t) = 0$. Thus, the first equation in (25) yields:

37 $$\begin{aligned} 0 = \left[ \hat{\mu }_{\mathrm{S}}(t) - \hat{\mu }_{\mathrm{S}}^{'}(t)\right] \nu _1(t). \end{aligned}$$

Taking into account (37), and by performing proper substitutions, (25) is transformed to the following DAE system:

38 $$\begin{aligned} \Sigma _{\mathrm{E}}^{(5)} :{\left\{ \begin{array}{ll} {\dot{\varepsilon }}_2(t) &{}= - u_1(t) \varepsilon _2(t) \\ {\dot{\varepsilon }}_3(t) &{}= - \left[ u_1(t) + u_2(t)\right] \varepsilon _3(t) \\ 0 &{}= \left[ \hat{\mu }_{\mathrm{S}}^{'}(t) - \hat{\mu }_{\mathrm{S}}(t) \right] \nu _1(t) \\ \varvec{\varepsilon }\left( t_0 \right) &{}= \varvec{\varepsilon }_0 \end{array}\right. }. \end{aligned}$$

Since the left-hand side of the third equation from (38) must be equal to zero $\forall t \in {\mathbb {T}}$, the following considerations about the observability/detectability of $\nu _2(t)$ are divided into two situations. The first situation takes place when the state variable $\nu _1(t)$ is always positive and both growth rate functions are the same, whereas the second is linked with the wash-out state. In this second situation, the state variable $\nu _1(t)$ becomes equal to zero either asymptotically or due to initial condition $\nu _1\left( t_0 \right) = 0$. Moreover, by assumption, both of the known inputs $u_1(t)$ and $u_2(t)$ are always positive. In the first situation, where $\nu _1(t)$ is always positive (and bounded by assumption), the right-hand side of the third equation in (38) is not equal to zero until $\hat{\mu }_{\mathrm{S}}(t) = \hat{\mu }_{\mathrm{S}}^{'}(t)$ $\forall t \in {\mathbb {T}}$. This leads to the conclusion that $\nu _2(t)$ must be strongly u-observable, what is imposed by $\varepsilon _2(t) = 0$ and ${\dot{\varepsilon }}_2(t) = 0$, $\forall t \in {\mathbb {T}}$. In the second situation, when the initial condition of the state variable $\nu _{1}(t)$ is equal to zero, the behaviour of the growth rate function strictly depends on changes of $\varepsilon _2(t)$. Thus, the first differential equation in (38) must be taken into account, which leads to the conclusion that $\nu _2(t)$ is globally strongly u-detectable. In turn, if the state variable $\nu _{1}(t)$ tends to zero asymptotically, the behaviour of the growth rate function also relies on changes of $\varepsilon _2(t)$. Hence, $\nu _2(t)$ is globally strongly u-detectable due to the same reasons where $\nu _1\left( t_0 \right) = 0$. Due to the fact that $\hat{\mu }_{\mathrm{S}}^{'}(t)$ and $\hat{\mu }_{\mathrm{S}}(t)$ are the Monod functions, if $\varepsilon _2(t) \rightarrow 0$, then $\hat{\mu }_{\mathrm{S}}^{'}(t) \rightarrow \hat{\mu }_{\mathrm{S}}(t)$ asymptotically.

To show the convergence of the growth rate functions, after assuming that $\forall t \in {\mathbb {T}} :~ \nu _1(t) \ne 0$, the following calculations are performed:

39 $$\begin{aligned} \begin{aligned} \dfrac{\nu _2(t)}{K_\mathrm{s}^0 + \nu _2(t)}&= \dfrac{\nu _2(t) - \varepsilon _2(t)}{K_\mathrm{s}^0 + \nu _2(t) - \varepsilon _2(t)} \\ \rightarrow ~ \left( K_\mathrm{s}^0 + \nu _2(t) \right) \left( \nu _2(t) - \varepsilon _2(t) \right)&= \nu _2(t) \left( K_\mathrm{s}^0 + \nu _2(t) - \varepsilon _2(t) \right) \rightarrow \\ K_\mathrm{s}^0\varepsilon _2(t)&= 0. \end{aligned} \end{aligned}$$

To meet the equality of both sides of (39), $\varepsilon _2(t)$ must be always equal to zero. This induces the observability of state variable $\nu _{2}(t)$.

The detectability of state variable $\nu _3(t)$ is associated with the fact that both known inputs of the system are always positive. Thus, it may be stated that the solution of the second differential equation in (38) tends to zero asymptotically. Therefore, in case (5), the state variable $\nu _2(t)$ is strongly u-observable, whereas the state variable $\nu _3(t)$ is strongly u-detectable.

An analogous analysis to cases (1) and (5) was carried out for the other combinations and its results, together with cases (1) and (5), are summarised in Table 3.

**Table 3:** Results of analysis based on indistinguishable dynamics without the uncertainty.

Case			Observability			Detectability		
No.	*y*(*t*)	$\hat{\mu }_{(\cdot )}(t)$	$\nu _1(t)$	$\nu _2(t)$	$\nu _3(t)$	$\nu _1(t)$	$\nu _2(t)$	$\nu _3(t)$
(1)	$\nu _3(t)$	$\hat{\mu }_{\mathrm{O}}(t)$	Yes	No	–	Yes	Yes	–
(2)	$\nu _1(t) > 0$	$\hat{\mu }_{\mathrm{O}}(t)$	-	No	Yes	–	Yes	Yes
	$\nu _1(t) = 0$ (IC)		–	No	No	–	Yes	Yes
	$\nu _1(t) = 0$ (A)		–	No	No	–	Yes	Yes
(3)	$\nu _2(t)$	$\hat{\mu }_{\mathrm{O}}(t)$	Yes	–	Yes	Yes	–	Yes
(4)	$\nu _3(t)$	$\hat{\mu }_{\mathrm{S}}(t)$	Yes	Yes	–	Yes	Yes	-
(5)	$\nu _1(t) > 0$	$\hat{\mu }_{\mathrm{S}}(t)$	–	Yes	No	–	Yes	Yes
	$\nu _1(t) = 0$ (IC)		–	No	No	–	Yes	Yes
	$\nu _1(t) = 0$ (A)		–	No	No	–	Yes	Yes
(6)	$\nu _2(t)$	$\hat{\mu }_{\mathrm{S}}(t)$	Yes	–	No	Yes	–	Yes
(7)	$\nu _1(t) > 0$	$\hat{\mu }_{\mathrm{SO}}(t)$	–	Yes	Yes	-	Yes	Yes
	$\nu _1(t) = 0$ (IC)		–	No	No	–	Yes	Yes
	$\nu _1(t) = 0$ (A)		–	No	No	–	Yes	Yes
(8)	$\nu _2(t)$	$\hat{\mu }_{\mathrm{SO}}(t)$	Yes	–	Yes	Yes	–	Yes
(9)	$\nu _3(t)$	$\hat{\mu }_{\mathrm{SO}}(t)$	Yes	Yes	-	Yes	Yes	-

### The summary of the observability/detectability analysis

Sections “Analysis based on differential geometry tools” and “Analysis based on the method of indistinguishable state trajectories” present an observability/detectability analysis of a non-linear system burdened by unknown inputs. First, the classic geometrical approach based on checking the invertibility of the observability matrix and checking the matching condition has been used. Secondly, the method based on the analysis of indistinguishable state trajectories has been employed. For nine cases, which include distinct choice of system measured outputs and form of the substitute reaction kinetics function, the research was performed in a way that if the first of the aforementioned methods reveals a negative result on state observability, the second method is applied and gives answers on state estimation possibility. Strong u-detectability has been proven for all cases, which entails asymptotic reconstruction of states and unknown input in the context of the measured output and the estimated reaction kinetics function. The analysis performed by using the indistinguishable dynamics method has also been applied in a situation when the uncertainty has been excluded. The comparison of results presented in Sections “Analysis for the CSTR model with uncertain dynamics” and “Analysis for the CSTR model without uncertain dynamics” shows how significantly the uncertainty occurrence affects the observability/detectability property. In fact, this analysis also shows that some approaches to system state (and also unknown input) estimation cannot be applied since full state observability cannot be obtained. It is worth mentioning that the results obtained by using the second method are non-trivial and entail performing certain transformations on DAE system dynamics to derive that a particular state variable (or unknown input) is observable/detectable or not. To make the above clearer, the conclusions drawn are summarised in Table 4. The symbols used in Table 4 stand for M—measured, O—observable, UO—unobservable, and D— detectable. It should be added that in the case of an approach based on the tools of differential geometry, the symbols ’O’ and ’UO’ have the meaning of meeting, or not the sufficient conditions of observability for the full considered model (without decomposition), respectively.

**Table 4:** Overview of the results of the observability/detectability analysis.

Case	Diff. geom.			Ind. dyn. + unc.			Ind. dyn. - unc.		
	$\nu _1(t)$	$\nu _2(t)$	$\nu _3(t)$	$\nu _1(t)$	$\nu _2(t)$	$\nu _3(t)$	$\nu _1(t)$	$\nu _2(t)$	$\nu _3(t)$
(1)	UO	UO	M	D	D	M	O	D	M
(2)	M	UO	UO	M	D	D	M	D	O/D
(3)	O	M	O	O	M	O	O	M	O
(4)	O	O	M	O	O	M	O	O	M
(5)	M	UO	UO	M	D	D	M	O/D	D
(6)	UO	M	UO	D	M	D	O	M	D
(7)	M	O	O	M	O	O	M	O/D	O/D
(8)	O	M	O	O	M	O	O	M	O
(9)	O	O	M	O	O	M	O	O	M

## Conclusions

In this paper, an observability/detectability analysis for selected non-linear uncertain model of biochemical processes with various sets of system measured outputs has been performed. As a model of the considered biochemical processes, a continuous stirred tank reactor with the microbial growth reaction and microbial mortality with the aggregated substrate and biomass concentrations in aerobic phase was used. In order to eliminate uncertainty from the system dynamics, it was transferred to an additional model component, which was then treated as an additional unknown input to the model. The analysis was performed for nine cases which covered a wide range of possible combinations of system measured outputs and unknown inputs. The observability/detectability properties were investigated using the classical approach based on differential geometry tools and the method of indistinguishable state trajectories (indistinguishable dynamics).

The delivered comprehensive analysis shows how the given structure of the mathematical model of the system is linked to the uncertainty of its dynamics, and how the selection of the system measured output affects the observability/detectability properties. The results obtained allowed to determine whether it is possible to develop a state observer, especially a sliding mode observer, depending on the available measured outputs of the considered system. Thus, these results, and the resulting conclusions may be used for state observer synthesis, and consequently for the development of monitoring and diagnostic systems for biochemical systems.

## Supplementary Information

Supplementary Information.

## Data Availability

All data generated or analysed during this study are included in this published article.
