# Supplementary material for: An observability and detectability analysis for non-linear uncertain CSTR model of biochemical processes
Source: Sci Rep. 2022 Dec 25;12:22327. doi: 10.1038/s41598-022-26656-3 (PMC9790892; doi:10.1038/s41598-022-26656-3)
Supplement: Supplementary file 1 — Supplementary Information. [file 41598_2022_26656_MOESM1_ESM.pdf]

Appendix A Idea of decomposition of system dynamics

The manifold $\boldsymbol{\mathcal{M}}$ of dimension n is divided into two sub-manifolds $\mathbb{X}_{\mathrm{m}} \subset \mathbb{R}^m$ and $\mathbb{H}_{\mathrm{b}} \subset \mathbb{R}^b$, which meet conditions of being disjoint manifolds, i.e., $\mathbb{X}_{\mathrm{m}} \cap \mathbb{H}_{\mathrm{b}} = \emptyset$. This leads to the following conclusions [1]:

1) $\boldsymbol{\mathcal{M}} = \mathbb{X}_{\mathrm{m}} \times \mathbb{H}_{\mathrm{b}} = \mathbb{X}_{\mathrm{m}} \oplus \mathbb{H}_{\mathrm{b}}$ - the Cartesian product of sub-manifolds is equal to direct sum of sub-manifolds.
2) $\mathrm{T}\boldsymbol{\mathcal{M}} = \mathrm{T}\mathbb{X}_{\mathrm{m}} \times \mathrm{T}\mathbb{H}_{\mathrm{b}} = \mathrm{T}\mathbb{X}_{\mathrm{m}} \oplus \mathrm{T}\mathbb{H}_{\mathrm{b}}$ - the Cartesian product of tangent bundles of sub-manifolds is equal to direct sum of tangent bundles of sub-manifolds.
3) $\mathrm{T}\mathbb{X}_{\mathrm{m}} \cap \mathrm{T}\mathbb{H}_{\mathrm{b}} = \emptyset$ - the tangent bundles of these sub-manifolds are disjoint set.

The new state variables of the decomposed system are defined as $\left[\boldsymbol{x}^{\mathrm{T}}(t)\ \boldsymbol{\xi}^{\mathrm{T}}(t)\right]^{\mathrm{T}} \triangleq \boldsymbol{\nu}(t)$ where $\boldsymbol{x}(t) \in \mathbb{X}_{\mathrm{m}} \subset \mathbb{R}^m$ and $\boldsymbol{\xi}(t) \in \mathbb{H}_{\mathrm{b}} \subset \mathbb{R}^b$. Moreover, taking into account some structural properties of the original system Σ_ν and assuming that the measuring equation is related only to the vector $\boldsymbol{x}(t)$, the decomposed system yields:

$$\Sigma_{\mathrm{d}_1}: \begin{cases} \dot{\boldsymbol{x}}(t) & = \boldsymbol{f}(\boldsymbol{x}(t), \boldsymbol{\xi}(t), t) + \sum\limits_{i_{\mathrm{p}}=1}^{p} \boldsymbol{b}_{i_{\mathrm{p}}}(\boldsymbol{x}(t), \boldsymbol{\xi}(t)) u_{i_{\mathrm{p}}}(t) \\ \dot{\boldsymbol{\xi}}(t) & = \boldsymbol{f}^{'}(\boldsymbol{x}(t), \boldsymbol{\xi}(t), t) + \sum\limits_{i_{\mathrm{p}}=1}^{p} \boldsymbol{b}^{'}_{i_{\mathrm{p}}}(\boldsymbol{x}(t), \boldsymbol{\xi}(t)) u_{i_{\mathrm{p}}}(t) \\ \boldsymbol{x}(t_0) & = \boldsymbol{x}_0 \\ \boldsymbol{\xi}(t_0) & = \boldsymbol{\xi}_0 \\ y(t) & = h(\boldsymbol{x}(t)) \end{cases}, \tag{A1}$$

where: $\forall t \in \mathbb{T}\colon\ \boldsymbol{f}\colon\ \mathbb{X}_{\mathrm{m}} \times \mathbb{H}_{\mathrm{b}} \times \mathbb{T} \to \mathrm{T}\mathbb{X}_{\mathrm{m}}$, $\forall t \in \mathbb{T}\colon\ \boldsymbol{f}^{'}\colon\ \mathbb{X}_{\mathrm{m}} \times \mathbb{H}_{\mathrm{b}} \times \mathbb{T} \to \mathrm{T}\mathbb{H}_{\mathrm{b}}$, $\forall t \in \mathbb{T}\colon\ \boldsymbol{b}_{i_{\mathrm{p}}}\colon\ \mathbb{X}_{\mathrm{m}} \times \mathbb{H}_{\mathrm{b}} \to \mathrm{T}\mathbb{X}_{\mathrm{m}}$, $\forall t \in \mathbb{T}\colon\ \boldsymbol{b}^{'}_{i_{\mathrm{p}}}\colon\ \mathbb{X}_{\mathrm{m}} \times \mathbb{H}_{\mathrm{b}} \to \mathrm{T}\mathbb{H}_{\mathrm{b}}$ are smooth maps whose connection with (1) is as follows:

$$\begin{aligned} \boldsymbol{\varphi}(\boldsymbol{\nu}(t), t) &\triangleq \left[\boldsymbol{f}^{\mathrm{T}}(\boldsymbol{x}(t), \boldsymbol{\xi}(t), t)\quad \boldsymbol{f}^{'\mathrm{T}}(\boldsymbol{x}(t), \boldsymbol{\xi}(t), t)\right]^{\mathrm{T}} \\ \boldsymbol{\vartheta}_{i_{\mathrm{p}}}(\boldsymbol{\nu}(t)) &\triangleq \left[\boldsymbol{b}^{\mathrm{T}}_{i_{\mathrm{p}}}(\boldsymbol{x}(t), \boldsymbol{\xi}(t))\quad \boldsymbol{b}^{'\mathrm{T}}_{i_{\mathrm{p}}}(\boldsymbol{x}(t), \boldsymbol{\xi}(t))\right]^{\mathrm{T}} \end{aligned}.$$

Due to uncertain nature of $\boldsymbol{\varphi}(\boldsymbol{\nu}(t), t)$, the new $\hat{\boldsymbol{\varphi}}(\boldsymbol{x}(t), \boldsymbol{\xi}(t))\colon\ \boldsymbol{\mathcal{M}} \to \mathrm{T}\boldsymbol{\mathcal{M}}$ being an exactly known (independent of parametric and unstructured uncertainty) assessment of $\boldsymbol{\varphi}(\boldsymbol{\nu}(t), t)$ is introduced:

$$\hat{\boldsymbol{\varphi}}(\boldsymbol{x}(t), \boldsymbol{\xi}(t)) \triangleq \left[\hat{\boldsymbol{f}}^{\mathrm{T}}(\boldsymbol{x}(t))\quad \hat{\boldsymbol{f}}^{'\mathrm{T}}(\boldsymbol{x}(t), \boldsymbol{\xi}(t))\right]^{\mathrm{T}},$$

where $\forall t \in \mathbb{T}\colon\ \hat{\boldsymbol{f}}\colon\ \mathbb{X}_{\mathrm{m}} \to \mathrm{T}\mathbb{X}_{\mathrm{m}}$, and $\forall t \in \mathbb{T}\colon\ \hat{\boldsymbol{f}}^{'}\colon\ \mathbb{X}_{\mathrm{m}} \times \mathbb{H}_{\mathrm{b}} \to \mathrm{T}\mathbb{H}_{\mathrm{b}}$ are smooth maps.

Taking the above into account, (A1) can be written as:

$$\Sigma_{\mathrm{d}_2}:\begin{cases}\dot{\boldsymbol{x}}(t) & =\hat{\boldsymbol{f}}(\boldsymbol{x}(t))+\sum_{i_{\mathrm{p}}=1}^{p}\boldsymbol{b}_{i_{\mathrm{p}}}(\boldsymbol{x}(t),\boldsymbol{\xi}(t))u_{i_{\mathrm{p}}}(t)\\ & +\boldsymbol{\rho}^{'}(\boldsymbol{x}(t))\Delta(\boldsymbol{x}(t),\boldsymbol{\xi}(t),t)\\ \dot{\boldsymbol{\xi}}(t) & =\hat{\boldsymbol{f}}^{'}(\boldsymbol{x}(t),\boldsymbol{\xi}(t))+\sum_{i_{\mathrm{p}}=1}^{p}\boldsymbol{b}^{'}_{i_{\mathrm{p}}}(\boldsymbol{x}(t),\boldsymbol{\xi}(t))u_{i_{\mathrm{p}}}(t)\\ & +\boldsymbol{\rho}^{''}(\boldsymbol{x}(t),\boldsymbol{\xi}(t))\Delta(\boldsymbol{x}(t),\boldsymbol{\xi}(t),t)\\ \boldsymbol{x}(t_0) & =\boldsymbol{x}_0\\ \boldsymbol{\xi}(t_0) & =\boldsymbol{\xi}_0\\ y(t) & =h(\boldsymbol{x}(t))\end{cases} \tag{A2}$$

where $\forall t\in\mathbb{T}$: $\boldsymbol{\rho}^{'}:\ \mathbb{X}_{\mathrm{m}}\to\mathrm{T}\mathbb{X}_{\mathrm{m}}$ and $\boldsymbol{\rho}^{''}:\ \mathbb{X}_{\mathrm{m}}\times\mathbb{H}_{\mathrm{b}}\to\mathrm{T}\mathbb{H}_{\mathrm{b}}$ are the smooth maps.

By defining $\boldsymbol{\alpha}(\boldsymbol{x}(t),\boldsymbol{\xi}(t))\triangleq\left[\boldsymbol{\rho}^{'\mathrm{T}}(\boldsymbol{x}(t))\ \ \boldsymbol{\rho}^{''\mathrm{T}}(\boldsymbol{x}(t),\boldsymbol{\xi}(t))\right]^{\mathrm{T}}$, the following phrase can be written:

$$\boldsymbol{\alpha}(\boldsymbol{x}(t),\boldsymbol{\xi}(t))\Delta(\boldsymbol{x}(t),\boldsymbol{\xi}(t),t)\triangleq\boldsymbol{\varphi}(\boldsymbol{x}(t),\boldsymbol{\xi}(t),t)-\hat{\boldsymbol{\varphi}}(\boldsymbol{x}(t),\boldsymbol{\xi}(t)). \tag{A3}$$

Component $\forall t\in\mathbb{T}$: $\Delta(\boldsymbol{x}(t),\boldsymbol{\xi}(t),t)\colon\ \mathbb{X}_{\mathrm{m}}\times\mathbb{H}_{\mathrm{b}}\times\underline{\mathbb{T}}\to\mathbb{W}\subset\mathbb{R}$ with $\|\Delta(\boldsymbol{x}(t),\boldsymbol{\xi}(t),t)\|=\max\{|\ \Delta(\boldsymbol{x}(t),\boldsymbol{\xi}(t),\underline{t})\ |: t\in\mathbb{T}\}\leq\overline{\Delta}<\infty$ may be considered as an additional bounded by $\overline{\Delta}$ input to system Σ_{d_2}. Moreover, taking into account (A3) and interpreting $\boldsymbol{\rho}^{'}$ and $\boldsymbol{\rho}^{''}$ as vectors parameterising the uncertainty of the dynamics of the original system (A1) the component $\boldsymbol{\alpha}(\boldsymbol{x}(t),\boldsymbol{\xi}(t))\Delta(\boldsymbol{x}(t),\boldsymbol{\xi}(t),t)$ models the parametric and unstructured uncertainty and thus represents (parameterised) unknown inputs of the system (A2). It makes the vector of state variables $\boldsymbol{x}(t)$ independent of the impact of uncertainty and vector $\boldsymbol{\xi}(t)$. In other words, the variables $\boldsymbol{\xi}(t)$ are not directly affecting the part of system dynamics, which is associated with state variables $\boldsymbol{x}(t)$. The unknown inputs expressed by $\Delta(\boldsymbol{x}(t),\boldsymbol{\xi}(t),t)$ are depended on $\boldsymbol{\xi}(t)$; however, they are not considered as tangent mapping but as certain distinct signals which coincide with the field $\boldsymbol{\alpha}(\boldsymbol{x}(t),\boldsymbol{\xi}(t))$. The idea of decomposition presented above is also illustrated in Fig. A1.

Appendix B Deliberation of observability of uncertain system in view of triangular observability form

In [2], by applying the method of indistinguishable state trajectories (indistinguishable dynamics), the authors presented an alternative approach to proving that a non-linear system with known (control) inputs in dynamics is (uniformly) observable, as established in [3]. By showing that the triangular observability form of 'error system' (8) dynamics is distinguishable,

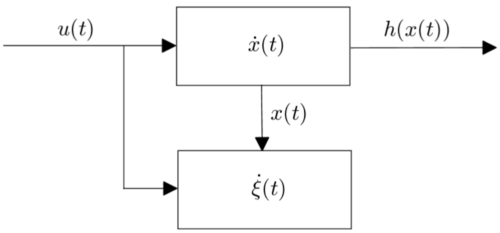

Fig. A1 The idea of decomposition of the considered system dynamics.

the 'observability map' (4) must be injective and its Jacobi matrix (6) must have full rank [4]. Taking into account that in [5–7] the system dynamics in the new coordinates is triangular with respect to known and unknown inputs, an interesting challenge arises. Namely, whether by using the method of indistinguishable state trajectories it is possible to prove that the conditions for the existence of an unknown input sliding mode observer hold. Thus, whether the equivalence of the considered methods for verifying the observability/detectability of a system with unknown inputs can be stated.

Let us introduce a triangular observability form for the MISO sub-class of affine system with unknown input (similar to (3)). After coordinate transformation $\tilde{\boldsymbol{\nu}}(t) = \boldsymbol{\Phi}(\boldsymbol{\nu}(t))$ between manifolds $\boldsymbol{\mathcal{M}}$ and $\tilde{\boldsymbol{\mathcal{M}}}$, the following is obtained:

$$\begin{cases} \dot{\tilde{\boldsymbol{\nu}}}(t) &= \tilde{\boldsymbol{A}}\tilde{\boldsymbol{\nu}}(t) + \tilde{\boldsymbol{\theta}}(\tilde{\boldsymbol{\nu}}(t), \boldsymbol{u}(t)) + \tilde{\boldsymbol{\rho}}(\tilde{\boldsymbol{\nu}}(t))\Delta(\boldsymbol{\nu}(t), t) \\ y(t) &= \tilde{\boldsymbol{C}}\tilde{\boldsymbol{\nu}}(t) \\ \tilde{\boldsymbol{\nu}}\,(t_0) &= \tilde{\boldsymbol{\nu}}_0 \end{cases}, \tag{B4}$$

where $\tilde{\boldsymbol{A}} \in \mathbb{R}^{(m+b)\times(m+b)}$, $\tilde{\boldsymbol{C}} \in \mathbb{R}^{1\times(m+b)}$ are the matrices of state and measured output, respectively, in canonical Brunovsky controllability form [8]:

$$\tilde{\boldsymbol{A}} = \begin{bmatrix} \boldsymbol{0}^{(m+b-1)\times 1} & \boldsymbol{I}^{(m+b-1)\times(m+b-1)} \\ 0 & \boldsymbol{0}^{1\times(m+b-1)} \end{bmatrix}, \ \tilde{\boldsymbol{C}} = \begin{bmatrix} 1 & 0 & \dots & 0 \end{bmatrix}. \tag{B5}$$

The smooth mappings $\tilde{\boldsymbol{\theta}}\colon\ \tilde{\boldsymbol{\mathcal{M}}} \times \mathbb{U}_{\mathrm{p}} \to T\tilde{\boldsymbol{\mathcal{M}}}$ and $\tilde{\boldsymbol{\rho}}\colon\ \tilde{\boldsymbol{\mathcal{M}}} \to T\tilde{\boldsymbol{\mathcal{M}}}$ are derived by utilising formulas presented in [5–7]. These mappings must meet:

$$\frac{\partial \tilde{\theta}_{i_{\mathrm{mb}}}}{\partial \tilde{x}_{i_{\mathrm{mb}}+1}} \neq 0, \ \frac{\partial \tilde{\rho}_{i_{\mathrm{mb}}}}{\partial \tilde{x}_{i_{\mathrm{mb}}+1}} \neq 0, \ i_{\mathrm{mb}} = \overline{1, m+b-1}, \tag{B6}$$

what is related to the invertibility property [2, 3, 6].

By invoking to the method of indistinguishable state trajectories presented in Sections 2.2 and 6.2, the 'error system' dynamics is derived as follows:

$$\begin{cases} \dot{\tilde{\varepsilon}}_1(t) &= \tilde{\varepsilon}_2(t) + \tilde{\theta}_1(\tilde{\nu}_1(t), \boldsymbol{u}(t)) + \tilde{\rho}_1(\tilde{\nu}_1(t))\Delta(\boldsymbol{\nu}(t), t) - \tilde{\theta}_1(\tilde{\nu}_1(t) \\ &\quad -\tilde{\varepsilon}_1(t), \boldsymbol{u}(t)) - \tilde{\rho}_1(\tilde{\nu}_1(t) - \tilde{\varepsilon}_1(t)) \left[\Delta(\boldsymbol{\nu}(t), t) - \delta\Delta(\boldsymbol{\nu}(t), \boldsymbol{z}(t), t)\right] \\ \dot{\tilde{\varepsilon}}_2(t) &= \tilde{\varepsilon}_3(t) + \tilde{\theta}_2(\tilde{\nu}_1(t), \tilde{\nu}_2(t), \boldsymbol{u}(t)) \\ &\quad +\tilde{\rho}_2(\tilde{\nu}_1(t), \tilde{\nu}_2(t))\Delta(\boldsymbol{\nu}(t), t) - \tilde{\theta}_2(\tilde{\nu}_1(t) - \tilde{\varepsilon}_1(t), \tilde{\nu}_2(t) - \tilde{\varepsilon}_2(t), \boldsymbol{u}(t)) \\ &\quad -\tilde{\alpha}_2(\tilde{\nu}_1(t) - \tilde{\varepsilon}_1(t), \tilde{\nu}_2(t) - \tilde{\varepsilon}_2(t)) \left[\Delta(\boldsymbol{\nu}(t), t) - \delta\Delta(\boldsymbol{\nu}(t), \boldsymbol{z}(t), t)\right] \\ &\quad \vdots \\ \dot{\tilde{\varepsilon}}_n(t) &= \tilde{\theta}_n(\tilde{\boldsymbol{\nu}}(t), \boldsymbol{u}(t)) + \tilde{\rho}_n(\tilde{\boldsymbol{\nu}}(t))\Delta(\boldsymbol{\nu}(t), t) - \tilde{\theta}_n(\tilde{\boldsymbol{\nu}}(t) - \tilde{\boldsymbol{\varepsilon}}(t), \boldsymbol{u}(t)) \\ &\quad -\tilde{\rho}_n(\tilde{\boldsymbol{\nu}}(t) - \tilde{\boldsymbol{\varepsilon}}(t)) \left[\Delta(\boldsymbol{\nu}(t), t) - \delta\Delta(\boldsymbol{\nu}(t), \boldsymbol{z}(t), t)\right] \\ 0 &= \tilde{\varepsilon}_1(t) \\ \tilde{\boldsymbol{\varepsilon}}(t_0) &= \tilde{\boldsymbol{\varepsilon}}_0 \end{cases}, \tag{B7}$$

where $\tilde{\boldsymbol{\varepsilon}}(t) \triangleq \tilde{\boldsymbol{\nu}}(t) - \tilde{\boldsymbol{z}}(t)$ and $\delta\Delta(\boldsymbol{\nu}(t), \boldsymbol{z}(t), t)$ are understood in the same way as their analogues in (24).

By imposing that $\forall t \in \mathbb{T}$: $\tilde{\varepsilon}_1(t) = 0$ and $\dot{\tilde{\varepsilon}}_1(t) = 0$ the components $\tilde{\theta}_1(\tilde{\nu}_1(t), \boldsymbol{u}(t))$ and $\tilde{\alpha}_1(\tilde{\nu}_1(t))$ are always equal to their analogues related to $\tilde{\boldsymbol{z}}(t)$. Hence, the first equation in (B7) transforms into:

$$\tilde{\varepsilon}_2(t) = -\tilde{\rho}_1(\tilde{\nu}_1(t))\delta\Delta(\boldsymbol{\nu}(t), \boldsymbol{z}(t), t). \tag{B8}$$

By invoking to proof presented in [2], all states $\tilde{\boldsymbol{\nu}}(t)$ are observable if all errors $\tilde{\boldsymbol{\varepsilon}}(t)$ are zero. In a situation when the unknown input $\Delta(\boldsymbol{\nu}(t), t)$ is not in the system dynamics, the subsequent errors $\tilde{\varepsilon}_{i_{\mathrm{mb}}}(t),\ i_{\mathrm{mb}} = \overline{2, m+b}$ become zero by recursive solving of all algebraic state derived equations of the triangular observability form established after the reduction of subsequent error derivatives. Taking into account that if the unknown input matching condition (7) must hold, then the mapping $\tilde{\rho}_1(\tilde{\nu}_1(t))$ cannot be equal to zero $\forall t \in \mathbb{T}$. Therefore, there arises a very interesting instance in a general view of observability/detectability analysis. To make $\tilde{\varepsilon}_2(t)$ equal to zero, and, what thereafter follows, for the observability of all state components $\delta\Delta(\boldsymbol{\nu}(t), \boldsymbol{z}(t), t)$ must be equal to zero. This indisputably leads to the conclusion that the uncertainty $\Delta(\boldsymbol{\nu}(t), t)$ is in some way able to be 'estimated'. This particular claim can be considered oppositely. If $\tilde{\varepsilon}_2(t)$ is always zero, then $\delta\Delta(\boldsymbol{\nu}(t), \boldsymbol{z}(t), t)$ must be undoubtedly too. Hence, the observability of all states and the unknown input is ensured. It means that the particular members of $\Delta(\cdot)$ must be distinguishable (and indistinguishable from themselves). According to the methodology presented in Section 6.2, the considered SMO input is by assumption able not only to estimate the unmeasured state but also to compensate/track the unknown input. Referring to [6, 7], when the first and second state variables

are 'ideally' tracked after reaching the sliding surface at certain time t_{reach}, then the sliding mode component 'injects' the same numerical value to the observer dynamics as the unknown input to the original dynamics. Due to the operation of the considered observer, the convergence of the unknown term estimates and the states is strictly related and takes place almost parallel in time. Therefore, a term occurs in the observer dynamics which provides elimination of mismatch between original and simplified dynamics, while all states and the unknown input are tracked. This provides to conclude that they are reconstructed. Thus, by invoking to the definition of UIO and its existence conditions [2, 9–11], when all states and unknown input in the model for analysis/synthesis purposes are at least u-detectable then they can be reconstructed. Hence, the observability conditions can be justified in the way of indistinguishable state trajectories.

Appendix C Observability analysis of the decomposed system

As mentioned in Section 6.1, the considered model is unobservable for some cases, i.a., case (1). However, in these cases, it is possible to decompose the system dynamics to obtain its observable part. Let us consider case (1) in detail. In this case, the second state variable $\nu_2(t)$ does not affect $\dot{\nu}_1(t)$ and $\dot{\nu}_3(t)$, but reverse dependence comes around. Also, both state variables $\nu_1(t)$ and $\nu_3(t)$ influence each other with dynamics $\dot{\nu}_1(t)$ and $\dot{\nu}_3(t)$, respectively. Thus, due to a proper choice of the reaction kinetics function is possible to ensure meeting the sufficient condition for observability of subsystem $(\nu_1(t), \nu_3(t))$. Hence, based on Appendix A, in case (1) $x_1(t) \triangleq \nu_1(t)$ and $x_2(t) \triangleq \nu_3(t)$, and $\xi(t) \triangleq \nu_2(t)$ is claimed as the parameter $\Delta(\boldsymbol{x}(t), \boldsymbol{\xi}(t), t)$. As a result, the new two-dimensional ($\nu_1(t)$ and $\nu_3(t)$ dependent) model for analysis purposes is obtained:

$$\hat{\boldsymbol{f}}(\boldsymbol{x}(t)) = \begin{bmatrix} \hat{\mu}_{\mathrm{O}}(t)x_1(t) - \beta_{\mathrm{m}}x_1(t) \\ -\frac{1}{Y_{\mathrm{o}}}\hat{\mu}_{\mathrm{O}}(t)x_1(t) - m_{\mathrm{o}}x_1(t) \end{bmatrix}, \ \boldsymbol{b}_1(\boldsymbol{x}(t)) = \begin{bmatrix} -x_1(t) \\ O_{\mathrm{in}} - x_3(t) \end{bmatrix},$$

$$\boldsymbol{b}_2(\boldsymbol{x}(t)) = \begin{bmatrix} 0 \\ O_{\mathrm{s}} - x_3(t) \end{bmatrix}, \ \boldsymbol{\rho} = \begin{bmatrix} 1 \\ -\frac{1}{Y_{\mathrm{o}}} \end{bmatrix},$$

$$\Delta(\boldsymbol{x}(t), \xi(t), t) = \left[\mu_{\mathrm{SO}}(t) - \hat{\mu}_{\mathrm{O}}(t)\right] x_1(t)$$

$$= \left[\mu_{\max}(t)\frac{\xi(t)}{K_{\mathrm{s}}(t)+\xi(t)}\frac{x_2(t)}{K_{\mathrm{o}}(t)+x_2(t)} - \mu^0_{\max}\frac{x_2(t)}{K^0_{\mathrm{o}}+x_2(t)}\right] x_1(t),$$

$$\hat{\boldsymbol{f}}^{'}(\boldsymbol{x}(t),\xi(t))=\left[-\frac{1}{Y_{\mathrm{s}}}\hat{\mu}_{\mathrm{O}}(t)x_1(t)-m_{\mathrm{s}}x_1(t)\right],\ \boldsymbol{b}_1^{'}(\xi(t))=\left[S_{\mathrm{in}}-\xi(t)\right],$$
$$\boldsymbol{b}_2^{'}=\left[0\right],\ \boldsymbol{\rho}^{'}=\left[-\frac{1}{Y_{\mathrm{s}}}\right], \tag{C9}$$
$$\xi\left(t_0\right)=\nu_2\left(t_0\right),\ x_1\left(t_0\right)=\nu_1\left(t_0\right),\ x_2\left(t_0\right)=\nu_3\left(t_0\right).$$

According to the presented methodology, by eliminating the second column and the second row from the original observability matrix (22), the new observability matrix of system (C9) yields:

$$\begin{aligned}\partial_{\boldsymbol{\nu}}\boldsymbol{\Phi}(\boldsymbol{x}(t))&=\begin{bmatrix}0&0&1\\ \star&0&\star\\ \star&0&\star\end{bmatrix}\\ \rightarrow\ \partial_{\mathrm{x}}\boldsymbol{\Phi}(\boldsymbol{x}(t))&=\begin{bmatrix}\partial_{\mathrm{x}}h\\ \partial_{\mathrm{x}}L_{\hat{\boldsymbol{f}}}h\end{bmatrix}=\begin{bmatrix}\partial_{x_1}h&\partial_{x_2}h\\ \partial_{x_1}L_{\hat{\boldsymbol{f}}}h&\partial_{x_2}L_{\hat{\boldsymbol{f}}}h\end{bmatrix}=\begin{bmatrix}0&1\\ \star&\star\end{bmatrix}\end{aligned}. \tag{C10}$$

Since the determinant of the observability matrix (C10) is equal to $\det\left[\partial_{\mathrm{x}}\boldsymbol{\Phi}(\boldsymbol{x}(t))\right]=-\star$, and if $\star\neq 0$, the rank of this matrix is 2 (except singular points). Thus, the subsystem '$\boldsymbol{x}(t)$' is locally observable.

References

[1] Spivak, M.: A Comprehensive Introduction to Differential Geometry. Vol. 1, 3rd edn. Publish or Perish, INC., Houston, Texas (1999)

[2] Moreno, J.A., Rocha-Cózatl, E., Vande Wouwer, A.: A dynamical interpretation of strong observability and detectability concepts for nonlinear systems with unknown inputs: application to biochemical processes. Bioproc. Biosyst. Eng. **37**(1), 37–49 (2014). https://doi.org/10.1007/s00449-013-0915-5

[3] Gauthier, J.P., Hammouri, H., Othman, S.: A simple observer for nonlinear systems applications to bioreactors. IEEE T. Automat. Contr. **37**, 875–880 (1992). https://doi.org/10.1109/9.256352

[4] Hermann, R., Krener, A.: Nonlinear controllability and observability. IEEE T. Automat. Contr. **22**, 728–740 (1977). https://doi.org/10.1109/TAC.1977.1101601

[5] Czyżniewski, M., Łangowski, R.: A robust sliding mode observer for non-linear uncertain biochemical. ISA T. **123**, 25–45 (2022). https://doi.org/10.1016/j.isatra.2021.05.040

[6] Veluvolu, K.C., Soh, Y.C.: Nonlinear Sliding Mode State and Unknown Input Estimations. VDM Verlag Dr. Muller, Saarbrucken, Germany (2009)

[7] Veluvolu, K.C., Soh, Y.C., Cao, W.: Robust observer with sliding mode estimation for nonlinear uncertain systems. IET Control Theory A. **1**, 1533–1540 (2007). https://doi.org/10.1049/iet-cta:20060434

[8] Isidori, A.: Nonlinear Control Systems. Springer, London, UK (1995)

[9] Ibarra-Rojas, S., Moreno, J.A., Espinosa-Pérez, G.: Global observability analysis of sensorless induction motors. Automatica **40**, 1079–1085 (2004). https://doi.org/10.1016/j.automatica.2004.01.020

[10] Moreno, J.A., Alvarez, J.: A bivalued observer for a class of uncertain reactors. IFAC Proceedings Volumes **46**, 261–266 (2013). https://doi.org/10.3182/20131216-3-IN-2044.00023

[11] Moreno, J.A., Alvarez, J.: On the estimation problem of a class of continuous bioreactors with unknown input. J. Process Contr. **30**, 34–49 (2015). https://doi.org/10.1016/j.jprocont.2014.12.005
